# Phytochemical Elucidation and Biological Activity Spectrum of *Rosmarinus officinalis* L.: Mechanistic Insights into the Antimicrobial, Antioxidant, and Apoptosis-Inducing Anticancer Effects of Carnosic Acid

**DOI:** 10.3390/metabo16070459

**Published:** 2026-06-30

**Authors:** Mohamed A. Fareid, Gamal M. El-Sherbiny, Nancy M. Elafandy, Nagat E. Eltoum, Mohamed S. Othman, Ahmad S. El-Hawary, Amr M. Shehabeldine, Fatma A. Hamada, Amira Salah El-Din Youssef

**Affiliations:** 1 Clinical Laboratory Science Department, Applied Medical Science College, University of Ha’il, Hail 2440, Saudi Arabia; m.alekhtaby@uoh.edu.sa (M.A.F.); n.elafandy@uoh.edu.sa (N.M.E.); 2 Botany and Microbiology Department, Faculty of Science, Al-Azhar University, Cairo 11884, Egypt; ahamdhawary@azhar.edu.eg (A.S.E.-H.); dramrshehab@azhar.edu.eg (A.M.S.); 3 Clinical Nutrition Department, Applied Medical Science College, University of Ha’il, Hail 2440, Saudi Arabia; ne.eltoum@uoh.edu.sa; 4 Biochemistry Department, College of Medicine, University of Ha’il, Hail 2440, Saudi Arabia; mo.abdelkarim@uoh.edu.sa; 5 Basic Sciences Department, First Year of Health and Medical Colleges, University of Ha’il, Hail 2440, Saudi Arabia; f.hamada@uoh.edu.sa; 6 Virology and Immunology Unit, Cancer Biology Department, National Cancer Institute, Cairo University, Cairo 11796, Egypt; amira.salah@nci.cu.edu.eg

**Keywords:** *R. officinalis*, carnosic acid, UHPLC/QTOF-MS, TLC, antimicrobial activity, biofilm inhibition, antioxidant activity, α-glucosidase inhibition, apoptosis, *BAX/BCL2*, MCF7 and HepG2 cells

## Abstract

**Background:** *Rosmarinus officinalis* L. is a medicinally important aromatic plant rich in bioactive secondary metabolites with diverse therapeutic properties. This study aimed to characterize the phytochemical profile of *R. officinalis* leaf extracts, isolate carnosic acid as a major bioactive diterpene, and evaluate its biological activities. **Methods**: Leaf extracts were prepared using solvents of increasing polarity and analyzed by phytochemical screening and UHPLC/QTOF-MS. Carnosic acid was isolated by thin-layer chromatography and assessed for antibacterial, antibiofilm, antioxidant, anti-inflammatory, antidiabetic, and antiproliferative activities using in vitro assays. Expression of apoptosis-related genes was also investigated. **Results:** Methanolic and ethanolic extracts exhibited the highest abundance of phenolic compounds and secondary metabolites, whereas the hexane extract showed lower phytochemical content. UHPLC/QTOF-MS identified seven major metabolites, including phenolic acids, flavonoids, and abietane-type diterpenes. Purified carnosic acid demonstrated potent antibacterial activity (MIC: 10–23 μg/mL) and inhibited biofilm formation by up to 90%. Strong antioxidant activity was observed, with DPPH and ABTS radical-scavenging IC_50_ values of 125 and 130 μg/mL, respectively. The compound also exhibited notable anti-inflammatory activity and markedly inhibited α-amylase and α-glucosidase activities. Furthermore, carnosic acid exhibited dose-dependent antiproliferative activity against MCF-7, HepG2, and MCF-10A cells, reducing cell viability to 10.8%, 16.9%, and 70.4 ± 1.8%, respectively, at 250 μg/mL, with corresponding IC_50_ values of 28.3, 37.8, and >250 μg/mL, respectively. Gene expression analysis revealed upregulation of *BAX* and downregulation of *BCL2*, indicating activation of mitochondrial-mediated apoptosis. **Conclusions:** *R. officinalis* leaves represent a valuable source of multifunctional phytochemicals, particularly carnosic acid. Its broad-spectrum biological activities and apoptosis-inducing potential support its promising application in pharmaceutical, nutraceutical, and biomedical fields.

## 1. Introduction

Plant secondary metabolites represent an exceptionally diverse and biologically active class of natural products that serve as a cornerstone for the discovery of novel therapeutic agents, functional foods, and nutraceutical formulations. Major phytochemical groups, including phenolic compounds, flavonoids, terpenoids, and alkaloids, have garnered extensive scientific attention because of their multifaceted pharmacological activities, encompassing antimicrobial, antioxidant, anti-inflammatory, anticancer, and metabolic regulatory effects. These compounds exert their biological functions through the modulation of multiple cellular signaling pathways, molecular targets, and biochemical networks, thereby offering significant potential for the prevention and treatment of complex multifactorial diseases. Consequently, comprehensive phytochemical profiling of medicinal plants, coupled with the isolation and characterization of their bioactive constituents, is essential for elucidating mechanisms of action, validating traditional therapeutic applications, and facilitating the development of evidence-based natural product–derived interventions [1,2].

Among medicinal and aromatic plants, *Rosmarinus officinalis* L. (rosemary), a perennial evergreen shrub belonging to the Lamiaceae family, occupies a prominent position due to its long-standing ethnomedicinal use and substantial industrial importance. Native to the Mediterranean basin and currently cultivated worldwide, rosemary is extensively utilized in the food, cosmetic, pharmaceutical, and nutraceutical sectors. In addition to its characteristic aroma and flavor, the plant has been traditionally employed for the management of inflammatory disorders, infectious diseases, digestive disturbances, and conditions associated with oxidative stress. The therapeutic efficacy of rosemary has been largely attributed to its chemically diverse phytoconstituent profile, which includes phenolic acids, flavonoids, triterpenes, essential oils, and phenolic diterpenes exhibiting potent biological activities [3,4].

Among the bioactive constituents identified in rosemary, carnosic acid has emerged as one of the most pharmacologically significant molecules. This lipophilic abietane-type phenolic diterpene constitutes a major antioxidant component of rosemary leaves and is recognized for its remarkable antioxidant, anti-inflammatory, antimicrobial, neuroprotective, anticancer, and metabolic regulatory activities. Mechanistically, carnosic acid functions as a redox-active molecule capable of modulating oxidative stress responses, inflammatory mediators, mitochondrial function, and apoptosis-related signaling pathways. These pleiotropic biological effects have positioned carnosic acid as a promising candidate for the development of therapeutic and preventive strategies targeting chronic inflammatory, neurodegenerative, metabolic, and malignant diseases [5,6,7].

Despite the extensive body of literature documenting the biological properties of rosemary and its constituents, important knowledge gaps remain regarding the relationship between extraction conditions, phytochemical composition, and biological activity. In particular, systematic investigations integrating solvent-dependent phytochemical variability with the efficient isolation, purification, and comprehensive functional evaluation of carnosic acid are relatively scarce. Most previous studies have primarily focused on rosemary essential oils or limited phytochemical analyses, frequently lacking comparative assessments of extraction efficiency using solvents of different polarities and their influence on the recovery of bioactive metabolites [6,7,8,9,10]. Such investigations are critical for optimizing extraction strategies and maximizing the therapeutic value of rosemary-derived compounds.

Furthermore, increasing experimental evidence has highlighted the anticancer potential of carnosic acid across a variety of malignancies. In hepatocellular carcinoma and other cancer models, carnosic acid has been shown to suppress tumor cell proliferation, migration, and invasion while inducing programmed cell death through the activation of both intrinsic and extrinsic apoptotic pathways. These effects are mediated by modulation of mitochondrial integrity, regulation of pro- and anti-apoptotic proteins, and activation of caspase cascades, including caspase-3, caspase-8, and caspase-9, ultimately leading to apoptotic cell death [11,12]. Such findings underscore the therapeutic promise of carnosic acid as a multifunctional anticancer agent and warrant further investigation into its molecular mechanisms and translational applications.

Importantly, the translational relevance of carnosic acid is strengthened by its well-established safety profile. The compound has been approved for use within the European Union as a natural food preservative and has received Generally Recognized As Safe (GRAS) status from the U.S. Food and Drug Administration (FDA). These regulatory endorsements reflect its favorable toxicological characteristics and support its continued development as a bioactive ingredient for pharmaceutical, nutraceutical, and functional food applications [13].

The polarity of the extraction solvent represents one of the most influential factors governing the efficiency of phytochemical recovery and the resulting chemical composition of plant-derived extracts. Variations in solvent polarity markedly affect both the qualitative and quantitative distribution of secondary metabolites by selectively solubilizing compounds according to their physicochemical characteristics. Highly polar solvents, such as methanol, are particularly effective in extracting hydrophilic phytoconstituents, including phenolic acids, flavonoids, and other polyphenolic compounds, which are frequently associated with antioxidant defense mechanisms, cellular redox homeostasis, and regulation of apoptosis-related signaling pathways. In contrast, nonpolar solvents such as hexane preferentially recover lipophilic metabolites, including terpenoids, sterols, and fatty acid derivatives, many of which participate in membrane-associated processes, mitochondrial function, and intracellular signaling cascades. Consequently, the application of solvent systems spanning a broad polarity range provides a comprehensive strategy for maximizing metabolite coverage and enables systematic assessment of solvent-dependent variations in phytochemical composition and biological activity [14,15].

Recent developments in high-resolution analytical technologies have substantially transformed phytochemical investigations, enabling unprecedented characterization of complex natural-product matrices. In particular UHPLC/QTOF-MS has emerged as a powerful platform for the sensitive detection, separation, and structural annotation of diverse classes of plant metabolites. The integration of UHPLC/QTOF-MS with sophisticated computational tools, including molecular networking and metabolomics-driven data analysis workflows, facilitates high-confidence identification of bioactive compounds, even within highly complex botanical extracts. These advances have significantly accelerated natural product discovery and enhanced understanding of the chemical diversity underlying medicinal plant bioactivity [16]. Concurrently, mechanistic investigations of purified phytochemicals have underscored the pivotal role of structure–activity relationships in determining biological efficacy, particularly with respect to anti-inflammatory, antioxidant, and anticancer properties, thereby providing critical insights into molecular mechanisms of action and therapeutic potential [3].

Despite extensive evidence supporting the antimicrobial, antioxidant, and anti-inflammatory activities of *R. officinalis* and its principal diterpene constituent, carnosic acid, the available literature remains fragmented and methodologically diverse. A considerable proportion of published studies have employed crude or only partially characterized extracts, frequently relying on limited phytochemical analyses and isolated biological endpoints. Moreover, many investigations lack comprehensive chemical standardization, rigorous statistical validation, and direct benchmarking against established reference compounds. Such methodological inconsistencies hinder meaningful comparisons among studies, limit mechanistic interpretation, and constrain the translational applicability of the reported findings [6,10].

Furthermore, a notable gap persists in the current body of knowledge due to the scarcity of integrated studies that combine advanced metabolomic profiling, targeted isolation of key bioactive metabolites, and multidimensional biological evaluation within a standardized experimental framework. In particular, the selective inhibitory activity of carnosic acid against α-glucosidase, together with its capacity to suppress biofilm formation at sub-minimum inhibitory concentrations (sub-MICs), remains insufficiently investigated despite their potential therapeutic significance in the management of metabolic disorders and microbial persistence.

In this context, the present study offers a comprehensive and integrated approach by combining UHPLC/QTOF-MS-based metabolomic characterization of *R. officinalis* leaf extracts with the isolation and structural identification of carnosic acid as a major bioactive constituent. Subsequently, the purified compound was subjected to systematic biological evaluation, with particular emphasis on its selective α-glucosidase inhibitory activity and antibiofilm effects at sub-MIC levels. Although the observed antioxidant, anti-inflammatory, antimicrobial, and antiproliferative activities are generally consistent with previous reports, the current investigation provides a unified, statistically validated, and experimentally standardized assessment of the multifunctional biological properties of carnosic acid. This integrated strategy strengthens the evidence base supporting its pharmacological relevance and further highlights its potential for future pharmaceutical, nutraceutical, and biomedical applications.

## 2. Materials and Methods

### 2.1. Chemicals, Reagents and Media

All chemicals, solvents, and reagents used in this study were of analytical or chromatographic grade and were used as received without further purification. Organic solvents, including hexane, ethyl acetate, methanol, and ethanol, together with gallic acid, quercetin, tannic acid, cholesterol, and the authentic carnosic acid reference standard, were purchased from Sigma-Aldrich (St. Louis, MO, USA). LC–MS-grade solvents and formic acid used for chromatographic and mass spectrometric analyses were obtained from Thermo Fisher Scientific (Waltham, MA, USA). Ammonium hydroxide, ammonium formate, and ammonium acetate, used for mobile-phase preparation and ionization optimization, were also supplied by Sigma-Aldrich (St. Louis, MO, USA). Ultrapure water was produced using a Milli-Q® water purification system (Merck Millipore, Bedford, MA, USA) and was used in all extraction, analytical, and bioassay procedures.

Microbiological culture media, including Mueller–Hinton broth (MHB) and Luria–Bertani (LB) broth, were purchased from HiMedia Laboratories Pvt. Ltd. (Mumbai, India). Established pharmaceutical standards were employed as positive controls for biological assays. Ciprofloxacin hydrochloride monohydrate (HiMedia Laboratories Pvt. Ltd., Mumbai, India) was used as the reference antibacterial agent. Diclofenac sodium (Sigma-Aldrich, St. Louis, MO, USA) served as the positive control in anti-inflammatory assays, acarbose (Sigma-Aldrich, St. Louis, MO, USA) was used as the standard α-glucosidase inhibitor in antidiabetic assays, and doxorubicin hydrochloride (Sigma-Aldrich, St. Louis, MO, USA) was employed as the reference chemotherapeutic agent in cytotoxicity and antiproliferative studies.

The exclusive use of certified analytical-grade reagents, high-purity solvents, and internationally recognized reference compounds ensured experimental rigor, analytical accuracy, and methodological reproducibility across all phytochemical, chromatographic, microbiological, biochemical, and cell-based analyses performed in the present study. Such standardization was essential for generating reliable, reproducible, and scientifically robust data suitable for comparative and translational research applications.

### 2.2. Collection Preparation of R. officinalis Leaves Extracts

#### 2.2.1. Plant Material Collection and Preparation

Fresh leaves of *R. officinalis* L. were procured from a commercial herbal market in Cairo, Egypt, during April 2025. The procurement, handling, and utilization of plant material were conducted in full compliance with applicable institutional, national, and international regulations governing plant research and the use of botanical resources. Authorization for the collection, identification, and scientific investigation of *R. officinalis* was formally granted by the Department of Botany and Microbiology, Faculty of Science, Al-Azhar University, Cairo, Egypt. All experimental procedures involving plant materials were performed in accordance with approved institutional policies and established ethical guidelines for botanical research.

Botanical authentication of the collected plant material was carried out by Dr. Shehata M. E., Department of Botany and Microbiology, Faculty of Science, Al-Azhar University, Cairo, Egypt. To ensure taxonomic traceability and reproducibility of the study, a representative voucher specimen was prepared, authenticated, and deposited in the Herbarium of the Department of Botany and Microbiology, Faculty of Science, Al-Azhar University. The specimen was assigned the accession number AZU/SCI/BOT/HERB/2026-12 and is maintained under standardized herbarium preservation conditions. The deposited voucher remains publicly accessible for future taxonomic verification, comparative studies, and reference purposes in accordance with the herbarium’s curation and access policies.

Following collection, the leaves were thoroughly washed with double-distilled water to eliminate surface-adhering particulate matter, dust, and other environmental contaminants. The cleaned plant material was then air-dried under controlled laboratory conditions at ambient temperature for 24 h to minimize thermal degradation of heat-sensitive phytochemicals. Subsequently, the dried leaves were finely pulverized using an electric laboratory grinder to obtain a uniform and homogeneous powder suitable for extraction and analytical procedures. The resulting powdered material was transferred into sterile, amber-colored airtight screw-cap containers and stored at −18 °C until further use.

These pre-analytical processing and storage procedures were carefully standardized to preserve the chemical integrity of secondary metabolites, prevent oxidative degradation and moisture-induced alterations, and ensure the stability and reproducibility of subsequent phytochemical, metabolomic, and biological investigations.

#### 2.2.2. Solvent Extraction Procedure

Extraction of *Rosmarinus officinalis* leaf powder was carried out using a conventional solvent maceration technique designed to facilitate the recovery of a broad spectrum of phytochemical constituents. Dried powdered leaf material (100 g) was extracted separately with 500 mL of analytical-grade hexane, ethyl acetate, methanol, or 95% (*v*/*v*) ethanol, corresponding to a plant material-to-solvent ratio of 1:5 (*w*/*v*). The selected solvents encompassed a wide polarity range to enable the efficient extraction of chemically diverse metabolites, including nonpolar lipophilic constituents and highly polar phenolic compounds.

The extraction process was performed in amber-colored glass vessels to minimize light-induced degradation of photosensitive metabolites. Maceration was conducted at room temperature (25 ± 2 °C) for 72 h with periodic agitation to enhance solvent penetration into plant tissues, promote diffusion of intracellular constituents, and improve mass-transfer efficiency. To maximize extraction recovery, the extraction mixtures were filtered at 24 h intervals through Whatman No. 1 filter paper, and the filtrates obtained from successive extraction cycles were pooled to generate representative crude extracts for each solvent system.

The combined filtrates were subsequently concentrated under reduced pressure using a rotary evaporator (Heidolph VV200, Schwabach, Germany) maintained at 45 °C. This controlled evaporation process was employed to efficiently remove extraction solvents while minimizing thermal decomposition and preserving the structural integrity of thermolabile phytochemicals. The concentrated extracts were further dried to constant weight to ensure complete solvent removal and facilitate accurate determination of extraction yield. Percentage extraction yields were calculated relative to the initial dry weight of the plant material.

The resulting dried crude extracts were transferred into sterile, airtight storage containers and maintained at 4 °C until subsequent phytochemical analyses, metabolomic profiling, compound isolation procedures, and biological activity evaluations. These standardized extraction and storage conditions were implemented to ensure optimal preservation of phytochemical stability, maintain extract quality, and enhance the reproducibility and reliability of downstream analytical and bioactivity assessments [17,18].

### 2.3. Quantitative Determination of Phytochemicals

The crude extracts of *R. officinalis* leaves obtained using hexane (HE), ethyl acetate (EAE), methanol (ME), and ethanol (EE) were comprehensively evaluated for their phytochemical composition through quantitative determination of major classes of secondary metabolites. The analysis focused on the estimation of total phenolic compounds, flavonoids, tannins, saponins, alkaloids, steroids, and terpenoids, which collectively represent key bioactive constituents responsible for the pharmacological properties of medicinal plants. Quantitative assessments were performed using validated spectrophotometric and gravimetric analytical procedures according to established protocols reported in the literature [16,19]. Spectrophotometric measurements were carried out using a UV–Visible spectrophotometer (UV-1800, Shimadzu Corporation, Kyoto, Japan) operated under standardized analytical conditions to ensure accuracy and consistency across all determinations. Calibration procedures and assay conditions were rigorously controlled to minimize analytical variability and enhance measurement reliability.

To ensure methodological robustness and reproducibility, each phytochemical assay was performed in triplicate using independently prepared samples. The resulting data were statistically analyzed and expressed as mean values ± standard deviation (SD), thereby providing a reliable representation of the quantitative distribution of secondary metabolites within the different solvent extracts. This comprehensive phytochemical characterization enabled comparative assessment of solvent-dependent extraction efficiency and facilitated correlation of metabolite abundance with subsequent biological activities.

#### 2.3.1. Total Saponin Content

Total saponin content (TSC) was determined using a gravimetric quantification procedure following the methodology reported by Fareid et al. [20], with minor modifications where appropriate. Briefly, aliquots (5 mL) of each extract solution at a predetermined concentration (mg/mL) were subjected to liquid–liquid extraction using *n*-butanol to selectively partition saponin constituents into the organic phase. Following phase separation, the pooled *n*-butanol fractions were washed repeatedly with 5% (*w*/*v*) sodium chloride solution to eliminate residual polar contaminants and non-saponin impurities, thereby enhancing the purity of the saponin-rich fraction.

The purified organic extract was subsequently concentrated under reduced pressure to remove the extraction solvent and then dried to constant mass to ensure complete solvent evaporation. The resulting saponin-enriched residue was accurately weighed using an analytical balance and used as the basis for quantitative determination.

Total saponin content was calculated by relating the mass of the recovered saponin-rich fraction to the initial dry weight of the extract and was expressed on a dry-weight basis. Quantitative values were determined according to the following equation:

$$\mathrm{Saponins}\;(\mathrm{mg}/g\;\mathrm{DW})=\frac{W}{C\times V}$$

where W represents the mass of the dried saponin-enriched residue recovered following solvent evaporation (mg), C denotes the concentration of the extract solution employed in the assay (mg/mL), and V corresponds to the volume of extract subjected to analysis (mL). The total saponin content was normalized to the dry weight of the extract and expressed as milligrams of saponins per gram of dry extract (mg/g DW). To ensure analytical accuracy, precision, and reproducibility, all measurements were conducted in triplicate using independently prepared samples. The obtained values were statistically evaluated and presented as mean ± standard deviation (SD).

#### 2.3.2. Total Alkaloid Content

Total alkaloid content (TAC) was quantified using a gravimetric precipitation method based on the procedure described by Fareid et al. [20]. Briefly, 5 mL aliquots of each extract solution at a known concentration (mg/mL) were treated with 2% (*v*/*v*) acetic acid in ethanol and incubated at ambient temperature for 4 h to ensure efficient solubilization and extraction of alkaloidal constituents. This acidic extraction step facilitated the conversion of alkaloids into their soluble salt forms, thereby enhancing extraction efficiency.

Following incubation, the mixtures were filtered to remove insoluble materials, and the resulting filtrates were concentrated under reduced pressure to decrease solvent volume and enrich the alkaloid-containing fraction. Subsequently, concentrated ammonium hydroxide was added gradually to the concentrated extracts until complete precipitation of alkaloids was achieved. The increase in pH promoted the conversion of soluble alkaloid salts into their free-base forms, resulting in the formation of a precipitated alkaloid fraction.

The precipitated alkaloids were collected by filtration, thoroughly washed with distilled water to eliminate residual reagents and co-extracted impurities, and then dried to constant mass to ensure complete removal of moisture. The dried alkaloid fraction was accurately weighed using an analytical balance and used for quantitative determination.

Total alkaloid content was calculated relative to the dry weight of the extract and expressed as milligrams of alkaloids per gram of dry extract (mg/g DW) according to the following equation:

$$\mathrm{Alkaloids}\;(\mathrm{mg}/g\;\mathrm{DW})=\frac{W_{2}-W_{1}}{C\times V}$$

where W_2_ represents the combined mass of the weighing container and the dried alkaloid residue (mg), and W_1_ denotes the mass of the empty container (mg). The net mass of the isolated alkaloid fraction was obtained by subtracting W_1_ from W_2_. C corresponds to the concentration of the extract solution employed in the assay (mg/mL), while V represents the volume of extract used for analysis (mL).

Total alkaloid content was normalized to the dry weight of the extract and expressed as milligrams of alkaloids per gram of dry extract (mg/g DW). All experimental measurements were conducted in triplicate using independently prepared samples to ensure methodological reliability and reproducibility. The resulting data were statistically analyzed and reported as mean ± standard deviation (SD).

#### 2.3.3. Total Tannin Content

Total tannin content (TTC) was quantified using a spectrophotometric Folin–Denis assay following the procedure described by Fareid et al. [20]. Briefly, aliquots (5 mL) of each extract solution at a defined concentration (mg/mL) were reacted with Folin–Denis reagent to initiate complex formation with phenolic constituents. Subsequently, sodium carbonate solution was added to provide an alkaline medium, thereby facilitating chromophore development. The reaction mixtures were incubated under controlled conditions to ensure complete color development prior to analysis.

Absorbance was recorded at 760 nm using a UV–Visible spectrophotometer, and tannin concentration was determined by reference to a calibration curve constructed using serial dilutions of tannic acid standard solutions under identical experimental conditions. The corresponding concentration derived from the calibration curve (C, mg/mL) was used for quantitative estimation. Total tannin content was normalized to the dry weight of the extract and expressed accordingly using the following equation:

$$\mathrm{Tannins}\;(\mathrm{mg}\;\mathrm{TAE}/g\;\mathrm{DW})=\frac{C\times V}{C_{e}\times V_{s}}$$

where C represents the tannic acid equivalent concentration derived from the calibration curve (mg/mL), V denotes the total reaction mixture volume (mL), C_e_ corresponds to the concentration of the extract solution used in the assay (mg/mL), and V_s_ indicates the volume of extract aliquot employed in the reaction system (mL).

Total tannin content was expressed as milligrams of tannic acid equivalents per gram of dry extract (mg TAE/g DW) following appropriate normalization to extract dry weight. All measurements were conducted in triplicate using independently prepared samples to ensure analytical robustness and reproducibility. The resulting data were statistically analyzed and reported as mean ± standard deviation (SD).

#### 2.3.4. Total Phenolic (TPC) and Flavonoid (TFC) Contents

Total phenolic content (TPC) and total flavonoid content (TFC) were determined using the Folin–Ciocalteu and aluminum chloride (AlCl_3_) colorimetric assays, respectively, as previously described [19], with minor methodological adaptations. For TPC determination, an aliquot of extract (0.5 mL) was reacted with 2.5 mL of 10% (*v*/*v*) Folin–Ciocalteu reagent, followed by the addition of 2.0 mL of 7.5% (*w*/*v*) sodium carbonate solution. The reaction mixture was incubated at ambient temperature for 30 min to allow complete chromophore development, after which absorbance was recorded at 765 nm using a UV–Visible spectrophotometer.

For TFC determination, 0.5 mL of each extract was mixed sequentially with 0.1 mL of 10% (*w*/*v*) aluminum chloride solution, 0.1 mL of 1 M potassium acetate, and 4.3 mL of distilled water. The reaction mixture was incubated for 30 min at room temperature to ensure stable complex formation between flavonoids and aluminum ions, and absorbance was subsequently measured at 415 nm.

Quantification of phenolic and flavonoid contents was performed using external calibration curves constructed with gallic acid (10–100 µg/mL) for TPC and quercetin (20–80 µg/mL) for TFC, respectively. The concentrations were calculated from the corresponding standard curves and expressed accordingly using the following equations:

$$\mathrm{TPC}\;(\mathrm{mg}\;\mathrm{GAE}/\mathrm{mL})=\frac{C_{\mathrm{GA}}\times V}{V_{s}},\mathrm{TFC}\;(\mathrm{mg}\;\mathrm{QE}/\mathrm{mL})=\frac{C_{Q}\times V}{V_{s}}$$

where C denotes the concentration determined from the respective calibration curve, V represents the total reaction volume, and V_s_ corresponds to the volume of extract aliquot used in the assay.

The calculated values were subsequently normalized to the dry weight of the extract to allow standardized comparison across samples and were expressed accordingly on a dry-weight basis.

$$\mathrm{TPC}\;\mathrm{or}\;\mathrm{TFC}\;(\mathrm{mg}/g\;\mathrm{DW})=\frac{\mathrm{Content}\;(\mathrm{mg}/\mathrm{mL})\times V_{\mathrm{extract}}}{\mathrm{mass}\;\mathrm{of}\;\mathrm{dry}\;\mathrm{extract}\;(g)}$$

#### 2.3.5. Total Steroid Content

Total steroid content (TSC) was determined using a spectrophotometric assay based on the method described by Fareid et al. [20], with slight modifications. Briefly, aliquots (0.5 mL) of each extract were reacted with acetic anhydride followed by the careful addition of concentrated sulfuric acid. The reaction mixtures were then incubated at ambient temperature for 15 min to allow complete development of the characteristic chromogenic complex.

Following incubation, absorbance was recorded at 620 nm using a UV–Visible spectrophotometer under standardized analytical conditions. Quantification was performed using an external calibration curve constructed with cholesterol standards within the concentration range of 20–80 µg/mL, prepared and analyzed under identical experimental conditions.

Steroid content was calculated using the corresponding calibration equation and expressed accordingly using the following formula:

$$\mathrm{Steroids}\;(\mathrm{mg}\;\mathrm{CE}/\mathrm{mL})=\frac{C\times V}{V_{s}}$$

where C = cholesterol concentration from the calibration curve (mg/mL), V = total reaction volume (mL), $V_{s}$ = volume of extract used (mL) To express results per gram of dry extract (DW):

$$\mathrm{Steroids}\;(\mathrm{mg}\;\mathrm{CE}/g\;\mathrm{DW})=\frac{\mathrm{Steroids}\;(\mathrm{mg}\;\mathrm{CE}/\mathrm{mL})\times V_{\mathrm{extract}}}{\mathrm{mass}\;\mathrm{of}\;\mathrm{dry}\;\mathrm{extract}\;(g)}$$

For reporting as a percentage of dry extract (% DW):

$$\mathrm{Steroids}\;(\%\;\mathrm{DW})=\frac{\mathrm{Steroids}\;(\mathrm{mg}\;\mathrm{CE}/g\;\mathrm{DW})}{10}$$

#### 2.3.6. Total Terpenoid Content

Total terpenoid content (TTC) was determined using a gravimetric approach based on solvent extraction followed by complete solvent removal, as described previously [20], with minor modifications. Following extraction and evaporation to dryness under controlled conditions, the resulting dried residue (W_t_) was collected and accurately weighed using an analytical balance.

The measured mass of the residual fraction was attributed to total terpenoid constituents, and quantification was subsequently performed on this basis. Terpenoid content was expressed relative to the dry weight of the extract using the following relationship:

$$\mathrm{Terpenoids}\;(\mathrm{mg}/\mathrm{mL})=\frac{W_{t}}{V}$$

where V is the volume of extract (mL). To express per gram of dry extract (DW):

$$\mathrm{Terpenoids}\;(\mathrm{mg}/g\;\mathrm{DW})=\frac{\mathrm{Terpenoids}\;(\mathrm{mg}/\mathrm{mL})\times V_{\mathrm{extract}}}{\mathrm{mass}\;\mathrm{of}\;\mathrm{dry}\;\mathrm{extract}\;(g)}$$

For reporting as a percentage of dry extract (% DW):

$$\mathrm{Terpenoids}\;(\%\;\mathrm{DW})=\frac{\mathrm{Terpenoids}\;(\mathrm{mg}/g\;\mathrm{DW})}{10}$$

All measurements were performed in triplicate, and results are reported as mean ± SD.

### 2.4. Chemical Analysis of the R. officinalis Methanolic Extract Using UHPLC/QTOF-MS

#### 2.4.1. Chemicals and Reagents

LC–MS-grade acetonitrile, along with gradient-grade organic solvents including isopropanol, methanol, dichloromethane, and ethyl acetate, were procured from Thermo Fisher Scientific (USA). Formic acid (≥98%), ammonium hydroxide, ammonium formate, and ammonium acetate were obtained from Sigma-Aldrich (St. Louis, MO, USA). All solvents and reagents employed in this study were of analytical or LC–MS grade quality and were used directly as supplied without any further purification or modification.

#### 2.4.2. Sample Preparation

The dried methanolic extract of *R. officinalis* (50 mg) was accurately weighed and reconstituted in 1000 µL of a solvent system comprising water, methanol, and acetonitrile in a ratio of 2:1:1 (*v*/*v*/*v*). The resulting solution was vortex-mixed for 2 min to ensure preliminary homogenization, followed by ultrasonication at 30 kHz for 10 min to achieve complete dissolution and enhance solubilization of phytoconstituents.

Subsequently, a 20 µL aliquot of the prepared stock solution (50 mg/1000 µL) was further diluted with 1000 µL of the same solvent mixture. The diluted sample was then centrifuged at 10,000 rpm for 5 min to remove particulate matter and insoluble residues. The clarified supernatant was filtered through a 0.22 µm PTFE syringe membrane filter to ensure particle-free sample preparation prior to instrumental analysis.

The filtrate was transferred into LC–MS-compatible vials, and 10 µL was injected for UHPLC/QTOF-MS analysis, corresponding to a final analytical concentration of 1 µg/µL. Blank samples and quality control (QC) samples were prepared using the mobile phase (water:methanol:acetonitrile, 2:1:1, *v*/*v*/*v*) and processed under identical experimental conditions to ensure analytical reproducibility, system stability, and data quality assurance. All samples were analyzed in both positive and negative electrospray ionization modes to ensure comprehensive detection and profiling of phytochemical constituents across diverse chemical classes [18].

#### 2.4.3. Instrumentation and UHPLC–QTOF-MS Conditions

For positive ionization mode, chromatographic separation was performed using a gradient elution system comprising solvent A (water supplemented with 0.1% formic acid) and solvent B (acetonitrile containing 0.1% formic acid). The gradient program was initiated at 90% solvent B, followed by a linear decrease to 10% B over 20 min to achieve efficient analyte separation. The mobile phase composition was then held at 10% B from 20 to 21 min, increased back to 90% B between 21 and 25 min, reduced again to 10% B from 25 to 28 min, and finally returned to 90% B to ensure adequate column re-equilibration and system stability.

In negative ionization mode, chromatographic separation was conducted under isocratic elution conditions using solvent C, consisting of methanol/water (95:5, *v*/*v*) supplemented with 5 mM ammonium acetate. These conditions were optimized to enhance retention behavior and ionization efficiency of acidic phytoconstituents, yielding well-resolved, symmetrical peaks and highly reproducible chromatographic performance while minimizing ion suppression and variability in ion response.

Mass spectrometric detection was carried out using a TripleTOF™ 5600+ high-resolution mass spectrometer (AB SCIEX, Concord, ON, Canada) equipped with a DuoSpray™ electrospray ionization (ESI) source. The ion spray voltage and declustering potential were set at 4500 V and 80 V in positive ion mode, and –4500 V and –80 V in negative ion mode, respectively. The source temperature was maintained at 600 °C, while curtain gas was adjusted to 25 psi. Nebulizer (Gas 1) and auxiliary (Gas 2) gases were both set to 40 psi to ensure optimal spray stability and desolvation efficiency. Collision energy was applied at 35 V (positive mode) and –35 V (negative mode), with a collision energy spread of 20 V and an ion tolerance threshold of 10 ppm to ensure accurate precursor–fragment ion matching.

Data acquisition was performed in information-dependent acquisition (IDA) mode, enabling simultaneous acquisition of high-resolution full-scan MS and MS/MS spectra. Survey scans were acquired across an *m*/*z* range of 50–1100 with a 50 ms accumulation time per scan. The most intense precursor ions detected in each survey scan were automatically selected for subsequent MS/MS fragmentation to generate structural information. All instrumental control, data acquisition, and batch processing were performed using Analyst TF software (version 1.7.1, AB SCIEX) [18,21].

#### 2.4.4. Data Processing and Compound Annotation

Non-targeted metabolomic profiling was performed using MS-DIAL software (version 3.70) for comprehensive feature extraction, deconvolution, and alignment of acquired LC–MS data. Metabolite annotation was carried out against the ReSpect spectral libraries, employing the appropriate ionization-specific databases for each analytical mode, including the positive-ion library (2737 entries) and the negative-ion library (1573 entries), in accordance with the acquisition polarity.

Data processing was conducted using optimized and standardized parameters to ensure high-resolution feature detection and accurate metabolite characterization. For peak picking and deconvolution, MS1 and MS2 mass accuracy tolerances were set at 0.01 Da and 0.05 Da, respectively. A minimum peak height threshold of 100 amplitude units was applied to reduce background noise, while a mass slice width of 0.05 Da was used to improve spectral resolution. Signal smoothing was performed across two consecutive scans, and a minimum peak width of six scans was enforced to ensure reliable feature definition.

For metabolite identification and alignment, MS1 and MS2 mass tolerances were set to 0.20 Da. Retention time alignment was carried out using a tolerance window of 0.05 min, while an MS1 alignment tolerance of 0.25 Da was applied to ensure accurate cross-sample feature matching. These parameters collectively enabled robust peak alignment and minimized false-positive feature assignments across datasets.

Putative metabolite annotations generated by MS-DIAL were subsequently validated using PeakView™ software (version 2.2) in conjunction with the MasterView™ (version 1.1) package (AB SCIEX, Framingham, MA, USA). Feature verification was performed through manual inspection of total ion chromatograms (TICs), applying stringent quality control criteria. Only signals exhibiting a signal-to-noise ratio (S/N) greater than 5 and sample-to-blank intensity ratios exceeding established thresholds, as recommended by Cajka and colleagues, were considered for final confirmation, thereby ensuring high confidence in metabolite identification and data reliability [21].

Metabolite identification was performed in strict accordance with the Metabolomics Standards Initiative (MSI) criteria for confidence levels in compound annotation. The majority of detected features were assigned to MSI Level 2, corresponding to putatively annotated metabolites based on high-resolution accurate mass measurements, characteristic isotopic distribution patterns, and diagnostic MS/MS fragmentation spectra. In contrast, carnosic acid was unambiguously identified at MSI Level 1 through direct comparison of its retention time, accurate mass, and fragmentation behaviour with an authenticated reference standard, thereby confirming its structural identity with highest confidence.

Due to the limited commercial availability of authentic reference standards for the broader set of detected metabolites, absolute quantification was not feasible for most compounds. Accordingly, metabolite levels were evaluated using a semi-quantitative approach based on normalized integrated peak areas derived from extracted ion chromatograms, enabling relative comparison of metabolite abundances across samples under standardized analytical conditions.

#### 2.4.5. Thin Layer Chromatography Analysis of Carnosic Acid

The presence and subsequent isolation of carnosic acid from the methanolic extract of Rosmarinus officinalis leaves were investigated using thin-layer chromatography (TLC) on pre-coated silica gel 60G plates (20 × 20 cm, 0.25 mm layer thickness; Merck, Darmstadt, Germany). Chromatographic separation was conducted using a nonpolar mobile phase composed of n-hexane and ethyl acetate in a ratio of 8:2 (*v*/*v*), selected to optimize resolution of lipophilic abietane-type diterpenoids.

Defined aliquots of the methanolic extract, alongside an authentic carnosic acid reference standard (Sigma-Aldrich, St. Louis, MO, USA), were applied as discrete application zones onto the TLC plates. The chromatographic development was carried out in a pre-saturated chamber under controlled conditions until the solvent front migrated to an appropriate distance, ensuring reproducible separation profiles.

Compound identification was achieved by direct comparative analysis of chromatographic migration behavior between the sample and the reference standard, with particular emphasis on retention factor (Rf) values and spot morphology. A distinct chromatographic band corresponding to carnosic acid was observed at an Rf value of 0.62, which was identical to that of the authentic standard, thereby providing strong confirmatory evidence for its presence in the methanolic extract of *R. officinalis* leaves [22].

The silica gel region corresponding to the carnosic acid band was carefully excised from the TLC plates and the adsorbed compound was subsequently recovered by elution with methanol. The obtained eluate was further subjected to repeated TLC-based purification cycles using freshly prepared silica gel 60G plates under identical chromatographic conditions to improve compound purity and to effectively remove potential co-migrating or structurally related impurities.

This preparative TLC purification strategy was performed across multiple independent chromatographic runs to ensure reproducibility and maximize recovery efficiency. The purified fractions obtained from successive runs were pooled and consolidated to yield a sufficiently enriched carnosic acid fraction, which was subsequently utilized for downstream UHPLC/QTOF-MS structural characterization as well as for comprehensive biological activity evaluation.

#### 2.4.6. Purity of Carnosic Acid

The TLC-isolated carnosic acid band was meticulously excised from the silica gel matrix, extracted using HPLC-grade methanol, and subsequently filtered to eliminate residual particulate silica. The resulting eluate was subjected to UHPLC/QTOF-MS analysis for definitive structural elucidation and confirmation.

Compound identity was established through comprehensive comparison of high-resolution analytical parameters, including exact mass accuracy, retention time consistency, isotopic pattern distribution, and diagnostic MS/MS fragmentation spectra, relative to an authenticated carnosic acid reference standard. This multi-parameter confirmation strategy unequivocally validated both the molecular identity and the high chemical purity of the isolated compound, thereby ensuring its suitability for downstream pharmacological and biological evaluations.

The purity of the isolated carnosic acid fraction was further assessed by UHPLC/QTOF-MS using a chromatographic peak area normalization approach. Briefly, the purified sample was dissolved in HPLC-grade methanol, passed through a 0.22 µm membrane filter to remove any residual particulates, and analyzed under identical UHPLC/QTOF-MS conditions as those applied for metabolite profiling.

Chromatographic data were processed using the instrument’s dedicated software platform, and purity was calculated as the proportional contribution of the carnosic acid peak area relative to the total integrated area of all detectable chromatographic signals within the chromatogram, according to the following equation:

$$\mathrm{Purity}\;(\%)=\left(\frac{\mathrm{Peak}\;\mathrm{area}\;\mathrm{of}\;\mathrm{carnosic}\;\mathrm{acid}}{\mathrm{Total}\;\mathrm{peak}\;\mathrm{area}\;\mathrm{of}\;\mathrm{all}\;\mathrm{detected}\;\mathrm{peaks}}\right)\times 100$$

A single predominant chromatographic peak corresponding to carnosic acid was detected at an approximate retention time of 23.4 min. This peak accounted for more than 95% of the total integrated chromatographic area, indicating a very high degree of chemical purity and confirming the successful isolation of the compound in substantially enriched form.

### 2.5. Antibacterial Activity of Purified Carnosic Acid

#### 2.5.1. Disc Diffusion Assay

The antibacterial activity of purified carnosic acid was assessed against a comprehensive panel of reference and foodborne bacterial strains, encompassing *Salmonella enterica* serovar Typhimurium (ATCC 35987), *Bacillus subtilis* (ATCC 6633), *Staphylococcus aureus* (ATCC 29213), *Pseudomonas aeruginosa* (ATCC 27853), *Klebsiella pneumoniae* (ATCC 4352), and *Escherichia coli* (ATCC 25922). All reference strains were obtained from the Bacteriology Laboratory, Department of Botany and Microbiology, Faculty of Science, Al-Azhar University, Cairo, Egypt, where they were routinely maintained under standardized culture conditions for experimental use. In addition, four foodborne clinical isolates, including *Staphylococcus aureus*, *Escherichia coli*, *Enterococcus faecalis*, and *Listeria monocytogenes*, obtained from the same institutional microbial collection, were included to evaluate the antimicrobial efficacy of carnosic acid against both clinically relevant and food-associated pathogenic bacteria.

Bacterial cultures were propagated in Mueller–Hinton broth (MHB; HiMedia Laboratories Pvt. Ltd., India) and incubated at 37 °C for 24 h. The resulting cultures were standardized to a 0.5 McFarland turbidity standard, corresponding to approximately 1.5 × 10^8^ CFU/mL. Aliquots (100 µL) of each standardized inoculum were uniformly spread onto Mueller–Hinton agar (MHA) plates using sterile cotton swabs to ensure homogeneous bacterial lawn formation.

Sterile filter paper discs were aseptically impregnated with 100 µL of purified carnosic acid solution (1 mg/mL in dimethyl sulfoxide, DMSO) and placed onto the inoculated agar surfaces. Ciprofloxacin (5 µg/disc) was used as a positive control for antibacterial efficacy, whereas discs containing DMSO alone served as negative controls to exclude solvent-related effects. Following incubation at 37 °C for 24 h under aerobic conditions, antibacterial activity was quantified by measuring the diameters of inhibition zones (IZD, mm) surrounding each disc. All assays were performed in triplicate under identical experimental conditions, and the results are expressed as mean ± standard deviation (SD) to ensure statistical reliability and reproducibility [23].

#### 2.5.2. Determination of Minimum Inhibitory Concentration

The MICs of ciprofloxacin hydrochloride monohydrate (HiMedia Laboratories Pvt. Ltd., India) and purified carnosic acid were determined using the broth microdilution technique in sterile 96-well microplates, in accordance with Clinical and Laboratory Standards Institute (CLSI) guidelines (M7-A7). Mueller–Hinton broth (MHB; HiMedia Laboratories Pvt. Ltd., India) was inoculated with standardized bacterial suspensions of *Salmonella enterica* serovar Typhimurium (ATCC 35987), *Bacillus subtilis* (ATCC 6633), *Staphylococcus aureus* (ATCC 29213), *Pseudomonas aeruginosa* (ATCC 27853), *Klebsiella pneumoniae* (ATCC 4352), and *Escherichia coli* (ATCC 25922), in addition to four foodborne clinical isolates (*Staphylococcus aureus*, *Escherichia coli*, *Enterococcus faecalis*, and *Listeria monocytogenes*), to achieve a final inoculum density of 1 × 10^6^ CFU/mL. Aliquots (200 µL) of the inoculated medium were dispensed into individual wells of the microplate.

Two-fold serial dilutions of ciprofloxacin (0.195–50 µg/mL) and carnosic acid (0.195–100 µg/mL) were prepared directly within the microplate wells to generate concentration gradients for susceptibility assessment. Negative control wells containing uninoculated MHB supplemented with the corresponding test compounds were included to evaluate background interference and ensure analytical validity. Following incubation at 37 °C for 18 h under aerobic conditions, bacterial growth was quantitatively assessed by measuring optical density at 630 nm using a microplate reader. The MIC was defined as the lowest concentration of the tested agent that completely inhibited visible bacterial growth, as evidenced by absence of turbidity.

For determination of the minimum bactericidal concentration (MBC), aliquots (10–20 µL) from wells exhibiting no detectable growth were aseptically subcultured onto Mueller–Hinton agar plates and incubated at 37 °C for 24 h. The MBC was defined as the lowest concentration resulting in no observable colony formation, corresponding to ≥99.9% reduction in the initial bacterial population. Antibacterial activity was further classified based on the MBC/MIC ratio, wherein values ≤ 4 were considered indicative of bactericidal effects, whereas values > 4 were interpreted as bacteriostatic activity. All experiments were performed in triplicate under identical conditions to ensure methodological robustness, reproducibility, and statistical reliability [19,24].

#### 2.5.3. Antibiofilm Properties of Purified Carnosic Acid

The antibiofilm potential of carnosic acid was evaluated using the crystal violet-based microtiter plate assay, as previously described by El-Sherbiny et al. [24], with minor methodological refinements. Bacterial strains as described above were cultured in Luria–Bertani (LB) broth (HiMedia Laboratories Pvt. Ltd., Thane, India) and standardized to an inoculum density of approximately 1 × 10^8^ CFU/mL. Aliquots (100 µL) of the standardized bacterial suspension were dispensed into sterile 96-well flat-bottom microplates containing serial two-fold dilutions of carnosic acid (0.39–9.0 µg/mL) prepared in LB broth.

The microplates were incubated under static conditions at 37 °C for 24 h to facilitate biofilm establishment on the abiotic surface. Following incubation, planktonic (non-adherent) cells were carefully aspirated, and the wells were gently washed three times with sterile phosphate-buffered saline (PBS) to remove loosely attached bacterial cells while preserving the adherent biofilm matrix.

The residual biofilms were subsequently fixed and stained with 0.1% (*w*/*v*) crystal violet solution for 15 min at room temperature. Excess dye was removed, and the wells were rinsed thoroughly with PBS to eliminate unbound stain. The retained crystal violet associated with the biofilm biomass was then solubilized using 100 µL of absolute ethanol per well. Biofilm formation was quantitatively assessed by measuring absorbance at 595 nm using a microplate reader (VMax, Molecular Devices, Sunnyvale, CA, USA).

Wells containing bacterial cultures without carnosic acid served as positive growth controls, whereas wells containing sterile culture medium alone were used as blank controls. The percentage inhibition of biofilm formation was calculated relative to the untreated control, enabling quantitative assessment of the antibiofilm efficacy of carnosic acid under standardized experimental conditions.

$$\mathrm{Biofilm}\;\mathrm{inhibition}\;(\%)=\left(\frac{A_{\mathrm{control}}-A_{\mathrm{sample}}}{A_{\mathrm{control}}}\right)\times 100$$

All experiments were performed in triplicate, and results were expressed as mean ± standard deviation (SD).

### 2.6. Antioxidant Activity of Purified Carnosic Acid

#### 2.6.1. DPPH Radical Scavenging Assay

The free radical scavenging capacity of purified carnosic acid was assessed using the 2,2-diphenyl-1-picrylhydrazyl (DPPH) assay in accordance with the method described by Fareid et al. [25], with minor modifications. Serial dilutions of carnosic acid were prepared within the concentration range of 7.81–1000 µg/mL. Subsequently, 100 µL of each test concentration was mixed with an equal volume (100 µL) of freshly prepared 0.1 mmol/L DPPH radical solution in methanol.

Ascorbic acid at corresponding concentrations was employed as the positive reference standard, whereas methanol served as the blank control. The reaction mixtures were incubated in the dark at 27 °C for 20 min to prevent photo-degradation of the DPPH radical and to ensure completion of the scavenging reaction. Following incubation, absorbance was recorded at 517 nm using a UV–Visible spectrophotometer.

The percentage of DPPH radical scavenging activity was calculated using the following equation:

$$\mathrm{DPPH}\;\mathrm{scavenging}\;\mathrm{activity}\;(\%)\;=\;[(A_{control}-\;A_{sample})/A_{control}]\times 100$$

where $A_{control}$ represents the absorbance of the DPPH solution in the absence of antioxidant (negative control), and $A_{sample}$ denotes the absorbance of the reaction mixture containing either carnosic acid or the reference standard.

The half-maximal inhibitory concentration (IC_50_), defined as the concentration required to scavenge 50% of DPPH radicals, was determined by plotting percentage radical scavenging activity against the logarithm of concentration, followed by non-linear regression analysis using GraphPad Prism software (Version 9.0; GraphPad Software LLC, San Diego, CA, USA). All experiments were conducted in triplicate, and results are presented as mean ± standard deviation (SD) to ensure statistical reliability and reproducibility.

#### 2.6.2. ABTS Radical Cation Decolorization Assay

The ABTS radical scavenging activity of purified carnosic acid was determined following the method described by Hui et al. [26], with minor modifications. The ABTS radical cation (ABTS) was generated by reacting a 7 mmol/L ABTS aqueous solution with 2.4 mmol/L potassium persulfate, followed by incubation in the dark at 25 °C for 12–16 h to ensure complete radical formation. Prior to analysis, the resulting ABTS working solution was diluted with ethanol (1:89, *v*/*v*) to obtain a stable absorbance of 0.70 ± 0.02 at 734 nm. Serial dilutions of carnosic acid and ascorbic acid (7.81–1000 µg/mL) were prepared and individually reacted with the diluted ABTS solution, while methanol was used as the blank control. After a defined incubation period, absorbance was recorded at 734 nm using a UV–Visible spectrophotometer, and the percentage of radical scavenging activity was calculated relative to the appropriate control. All experiments were conducted in triplicate, and the results are expressed as mean ± standard deviation (SD) derived from three independent experimental replicates to ensure analytical precision and reproducibility.

### 2.7. Anti-Inflammatory Activity

The anti-inflammatory potential of purified carnosic acid was assessed using the human red blood cell (HRBC) membrane stabilization assay, following the methodology described by Elbestawy et al. [18], with appropriate experimental standardization. Fresh peripheral blood was obtained from a healthy adult volunteer who had not received any nonsteroidal anti-inflammatory drugs (NSAIDs) for at least one week prior to sample collection. Written informed consent was obtained prior to venipuncture. The study protocol was reviewed and approved by the Institutional Review Board (IRB) of the National Cancer Institute, Cairo University, Egypt (IRB No.: IRB-00004025; Approval No.: CB2410-102-083-192, 2024), and all procedures were conducted in strict accordance with national ethical regulations and the principles outlined in the Declaration of Helsinki.

Collected blood was immediately mixed with Alsever’s solution in a 1:1 ratio (comprising 2% dextrose, 0.8% sodium citrate, 0.5% citric acid, and 0.42% sodium chloride) to prevent coagulation and preserve erythrocyte integrity. The mixture was centrifuged at 3000 rpm, and the erythrocyte pellet was washed repeatedly (three cycles) with isotonic saline solution (0.9% NaCl) to remove plasma residues and other contaminants. The purified erythrocytes were then resuspended in isotonic saline to obtain a standardized 10% (*v*/*v*) HRBC suspension.

Serial dilutions of carnosic acid (7.81–1000 µg/mL) were prepared in dimethyl sulfoxide (DMSO) for evaluation of dose-dependent activity. Sodium diclofenac (Sigma-Aldrich, St. Louis, MO, USA) at corresponding concentrations was used as the reference anti-inflammatory agent, while DMSO served as the negative control.

For each experimental setup, 1 mL of phosphate buffer, 2 mL of hypotonic saline, and 0.5 mL of HRBC suspension were combined with the test or control solutions. The reaction mixtures were incubated at 37 °C for 30 min to induce hypotonic stress, followed by centrifugation at 3000 rpm for 20 min to separate intact cells from released hemoglobin. The extent of hemolysis was quantified spectrophotometrically by measuring hemoglobin release in the supernatant at 560 nm, using isotonic saline as the blank. The percentage inhibition of hemolysis, reflecting membrane stabilization and anti-inflammatory activity, was calculated using the standard equation provided below:

$$\mathrm{Inhibition}\;\mathrm{of}\;\mathrm{hemolysis}\;(\%)=\frac{A_{control}-A_{sample}}{A_{control}}\times 100$$

where $A_{control}$ and $A_{sample}$ represent the absorbance of the control and the test sample, respectively. The IC_50_ values were determined by plotting the percentage inhibition against the logarithm of concentration and calculating the concentration corresponding to 50% inhibition using nonlinear regression analysis (GraphPad Prism).

### 2.8. Antidiabetic Activity

#### 2.8.1. α-Amylase Inhibition Assay

The α-amylase inhibitory activity of purified carnosic acid was evaluated using a modified protocol based on Fareid et al. [25]. Briefly, serial dilutions of carnosic acid (7.81–1000 µg/mL) were pre-incubated with 500 µL of 0.02 M sodium phosphate buffer (pH 6.9, supplemented with 0.006 M NaCl) together with porcine pancreatic α-amylase (0.5 mg/mL; Sigma-Aldrich, Cat. No. A3176) at 25 °C for 10 min to allow enzyme–inhibitor interaction. Acarbose at equivalent concentrations was employed as a positive reference inhibitor. Following pre-incubation, 500 µL of 1% (*w*/*v*) soluble starch prepared in the same buffer was added as substrate, and the reaction mixture was further incubated for 10 min under controlled conditions to permit enzymatic hydrolysis.

The reaction was terminated by the addition of 1.0 mL of 96 mM 3,5-dinitrosalicylic acid (DNS) reagent, followed by incubation in a boiling water bath for 5 min to develop the chromogenic product. After cooling to room temperature, the reaction mixture was diluted with 10 mL of distilled water to stabilize the color intensity, and absorbance was recorded at 450 nm using a UV–Visible spectrophotometer. A reagent blank containing buffer and DNS solution without enzyme or substrate was included to correct for baseline absorbance and ensure analytical accuracy.

#### 2.8.2. α-Glucosidase Inhibition Assay

The α-glucosidase inhibitory activity of purified carnosic acid was assessed using a microplate-based enzymatic assay as previously described [25], with minor modifications. Serial dilutions of carnosic acid and acarbose (7.81–1000 µg/mL) were prepared and incubated with 100 µL of 0.1 M sodium phosphate buffer (pH 6.9) containing yeast-derived α-glucosidase (1.0 U/mL; Sisco Research Laboratories Pvt. Ltd., Mumbai, India) in sterile 96-well microplates. The reaction mixtures were pre-incubated at 25 °C for 10 min to facilitate enzyme–inhibitor interaction.

Subsequently, 50 µL of 5 mM p-nitrophenyl-α-D-glucopyranoside (pNPG; Sigma-Aldrich, Cat. No. N7006) prepared in the same buffer was added as the chromogenic substrate, and incubation was continued for an additional 5 min at 25 °C under controlled conditions. Enzymatic hydrolysis was monitored by measuring absorbance at 405 nm using a microplate reader (VMax, Molecular Devices, Sunnyvale, CA, USA), with readings taken before and after incubation to determine enzymatic activity.

Control wells containing buffer in place of test compound were included to correct for baseline enzyme activity. The percentage inhibition of α-glucosidase activity was calculated using the standard equation provided below.

$$\mathrm{Inhibition}\;(\%)=\frac{\left(A_{\mathrm{sample}}-A_{\mathrm{sample}\;\mathrm{blank}}\right)}{\left(A_{\mathrm{control}}-A_{\mathrm{control}\;\mathrm{blank}}\right)}\times 100$$

#### 2.8.3. Anticancer Activity of Carnosic Acid

The in vitro anticancer activity of purified carnosic acid was assessed using the MTT colorimetric assay, as previously described by Abdel Fattah et al. [27], with minor methodological refinements. Human breast adenocarcinoma (MCF-7), hepatocellular carcinoma (HepG2), and normal human breast epithelial (MCF-10A) cell lines were obtained from the American Type Culture Collection (ATCC, Rockville, MD, USA) and cultured under standard conditions. Cells were seeded into sterile 96-well plates and incubated overnight to facilitate adherence and establishment of a confluent monolayer.

Following attachment, the growth medium was replaced with fresh medium containing graded concentrations of carnosic acid (6.25–250 µg/mL), and cells were further incubated at 37 °C for 24 h under controlled humidified conditions (5% CO_2_). Untreated cells served as negative controls to define baseline viability. After exposure, cells were gently washed three times with cold phosphate-buffered saline (PBS) to remove residual test compound and culture medium.

Subsequently, MTT reagent (0.5 mg/mL) was added to each well and incubated for 2–5 h at 37 °C, allowing metabolically active cells to reduce the tetrazolium salt into insoluble formazan crystals. Following incubation, the supernatant was carefully aspirated, and 200 µL of dimethyl sulfoxide (DMSO) was added to solubilize the formed formazan product.

Absorbance was measured at 570 nm using a microplate reader, and cellular metabolic activity was quantified based on optical density values. Cell viability and cytotoxic effects were calculated using standard mathematical formulations derived from absorbance ratios relative to untreated control cells.

$$\mathrm{Cell}\;\mathrm{viability}\;(\%)=\left(\frac{{\mathrm{OD}}_{\mathrm{treated}}}{{\mathrm{OD}}_{\mathrm{control}}}\right)\times 100,\mathrm{Cell}\;\mathrm{death}\;(\%)=\left(\frac{{\mathrm{OD}}_{\mathrm{control}}-{\mathrm{OD}}_{\mathrm{treated}}}{{\mathrm{OD}}_{\mathrm{control}}}\right)\times 100$$

#### 2.8.4. Effects of Carnosic Acid on Apoptosis-Related Gene Expression

MCF-7 and HepG2 cells were exposed to carnosic acid (25 µg/mL) for 24 h, alongside corresponding untreated control groups. Doxorubicin (Sigma-Aldrich, USA; 5 µg/mL) was included as a positive control to validate cytotoxic and apoptosis-inducing responses. Following treatment, total RNA was isolated from both experimental and control samples using a commercial RNA extraction kit (Thermo Fisher Scientific, USA) in accordance with the manufacturer’s protocol.

RNA yield and purity were evaluated spectrophotometrically using a NanoDrop system, and only samples exhibiting an A260/A280 ratio of approximately 1.8 were considered suitable for downstream molecular analyses. First-strand complementary DNA (cDNA) synthesis was performed using 1 µg of total RNA with a reverse transcription system incorporating both random hexamer and oligo(dT) primers, as per the supplier’s instructions.

Quantitative real-time PCR (qRT-PCR) was conducted using SYBR Green chemistry on a real-time thermal cycler to assess the expression profiles of the pro-apoptotic gene *BAX* and the anti-apoptotic gene *BCL2*, with *GAPDH* employed as the internal reference gene for normalization. Primer sequences are listed in Table 1.

Thermal cycling conditions consisted of an initial denaturation step at 95 °C for 10 min, followed by 40 amplification cycles comprising denaturation at 95 °C for 15 s, annealing at 59–60 °C for 40 s, and extension at 72 °C for 20 s. Relative gene expression levels were quantified using the comparative threshold cycle (2^−ΔΔCt^) method and expressed as fold changes relative to untreated controls after normalization to *GAPDH* expression [28].

### 2.9. Statistical Analysis

All experiments were conducted in biological and technical triplicates (*n* = 3), and the obtained data are presented as mean ± standard deviation (SD) to ensure statistical reliability and reproducibility. Statistical analyses were performed using one-way and two-way analysis of variance (ANOVA), as appropriate, followed by Tukey’s post hoc multiple comparison test to identify statistically significant differences among experimental groups.

Where applicable, comparisons between two independent groups were additionally evaluated using an unpaired Student’s *t*-test. A probability value of *p* < 0.05 was considered indicative of statistical significance across all analyses.

## 3. Results

### 3.1. Extraction and Phytochemical Analysis of R. officinalis Leaves

Powdered leaves of *R. officinalis* (100 g) were subjected to sequential extraction using solvents of progressively increasing polarity, namely hexane, ethyl acetate, methanol, and ethanol (500 mL each; 24 h; room temperature). The corresponding extraction yields were 4.5%, 5.2%, 7.1%, and 6.2% (*w*/*w*, dry weight basis), respectively, demonstrating a clear solvent-dependent variability in extraction efficiency and metabolite recovery profiles.

Overall, polar solvents, particularly methanol and ethanol, exhibited markedly enhanced extraction efficiency for secondary metabolites, including phenolic compounds, flavonoids, alkaloids, and saponins, compared with intermediate- and non-polar solvents (ethyl acetate and hexane). This trend highlights the preferential solubilization of hydrophilic and semi-polar phytoconstituents in polar media.

Comprehensive quantitative phytochemical profiling of the hexane (HE), ethyl acetate (EAE), methanol (ME), and ethanol (EE) extracts confirmed the presence of multiple bioactive metabolite classes, including saponins, alkaloids, tannins, flavonoids, phenolics, steroids, and terpenoids (Table 2), with significant variability driven by solvent polarity and differential compound solubility.

Saponins were detected across all extract fractions, with maximal accumulation observed in the methanolic extract (35.0 ± 1.2 mg/g DW), followed by ethanol (32.0 ± 1.0 mg/g DW), whereas the hexane fraction exhibited the lowest concentration (12.5 ± 0.5 mg/g DW). A similar distribution pattern was observed for alkaloids, which were most abundant in ME (27.5 ± 1.0 mg/g DW) and EE (25.0 ± 0.8 mg/g DW), and minimal in HE (8.0 ± 0.3 mg/g DW).

Total phenolic (45.0 ± 1.5 mg GAE/g DW) and tannin contents (32.0 ± 1.1 mg TAE/g DW) were significantly enriched in the methanolic extract, while hexane consistently yielded the lowest levels, reflecting the limited solubility of polar phytochemicals in nonpolar solvents. Flavonoids, well recognized for their antioxidant and antimicrobial properties, were similarly concentrated in ME (40.0 ± 1.3 mg QE/g DW) and EE (38.0 ± 1.2 mg QE/g DW).

Notably, steroids and terpenoids, despite their predominantly lipophilic nature, were also relatively enriched in polar extracts, with terpenoid content reaching 41.0 ± 1.4 mg/g DW in ME, suggesting possible co-extraction phenomena mediated by solvent–matrix interactions and compound association effects.

Statistical analysis using one-way ANOVA followed by Tukey’s post hoc test revealed significant differences (*p* < 0.05) among solvent fractions across all evaluated phytochemical classes, including saponins, alkaloids, tannins, flavonoids, and total phenolics. Post hoc comparisons further confirmed that methanol and ethanol extracts were significantly enriched in these bioactive constituents relative to the hexane fraction, while ethyl acetate exhibited intermediate extraction behavior depending on metabolite class.

Collectively, these findings demonstrate that solvent polarity is a critical determinant of both extraction efficiency and phytochemical distribution in *R. officinalis* leaves, with methanol and ethanol representing the most effective solvents for the recovery of polar and semi-polar bioactive metabolites.

### 3.2. UHPLC/QTOF-MS Profiling of R. officinalis Methanolic Extract

**Figure 1:**
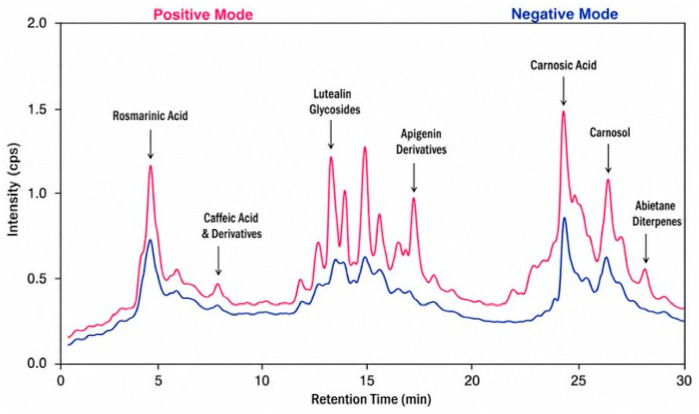
LC–MS chromatograms of the sample obtained in positive ionization mode (ESI^+^) and negative ionization mode (ESI^−^), showing intensity (counts per second, cps) versus retention time (min).

Ultra-high-performance liquid chromatography coupled with quadrupole time-of-flight mass spectrometry was utilized for comprehensive phytochemical characterization of the methanolic extract of *R. officinalis* leaves. The total ion chromatogram (TIC) exhibited a chemically complex metabolite landscape, with well-resolved chromatographic features spanning a broad retention time range (approximately 3–30 min), indicative of a diverse array of constituents varying in polarity, structural class, and physicochemical properties, as depicted in Figure 1. and summarized in Table 3. Early-eluting peaks (approximately 4–6 min) were predominantly associated with low-molecular-weight phenolic acids, with rosmarinic acid identified as a major discriminating feature. Additional signals detected within the 6–9 min retention window were attributed to caffeic acid and structurally related phenylpropanoid derivatives. The intermediate retention interval (approximately 13–17 min) was characterized by dense and overlapping peak clusters corresponding primarily to flavonoid glycosides, including luteolin- and apigenin-derived conjugates, reflecting the high abundance of oxygenated polyphenolic scaffolds within the extract matrix. Late-eluting constituents (approximately 22–28 min) were largely assigned to lipophilic diterpenoid compounds, most notably carnosic acid and its oxidized analogue carnosol, along with other abietane-type diterpenes, consistent with their increased hydrophobicity and stronger interaction with the stationary phase. In negative electrospray ionization mode (ESI^−^), carnosic acid was confidently identified based on the presence of its deprotonated molecular ion [M–H]^−^ at *m*/*z* 331, corresponding to the molecular formula C_20_H_28_O_4_. Tandem mass spectrometric analysis further revealed characteristic fragment ions at *m*/*z* 317 and 299, providing strong structural corroboration. In parallel, the ion at *m*/*z* 329 was assigned to carnosol, in agreement with the compound annotations presented in Table 3.

### 3.3. Thin-Layer Chromatographic Purification and Identification of Carnosic Acid

**Figure 2:**
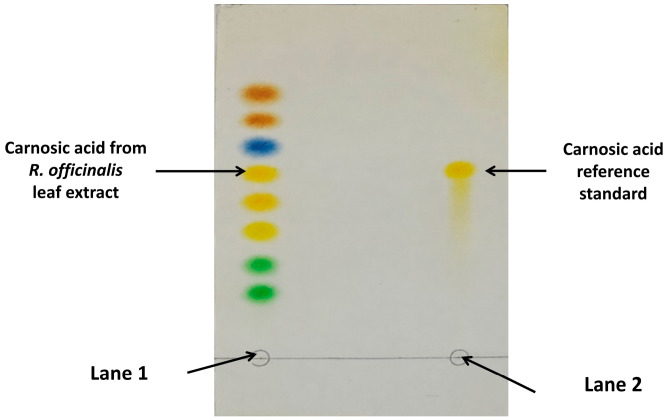
Thin-layer chromatographic purification of carnosic acid from *R. officinalis* leaf methanolic extract. Lane 1: methanolic extract showing multiple resolved bands. Lane 2: authentic carnosic acid standard, co-migrating with the corresponding band in the extract, confirming the identity of carnosic acid.

**Figure 3:**
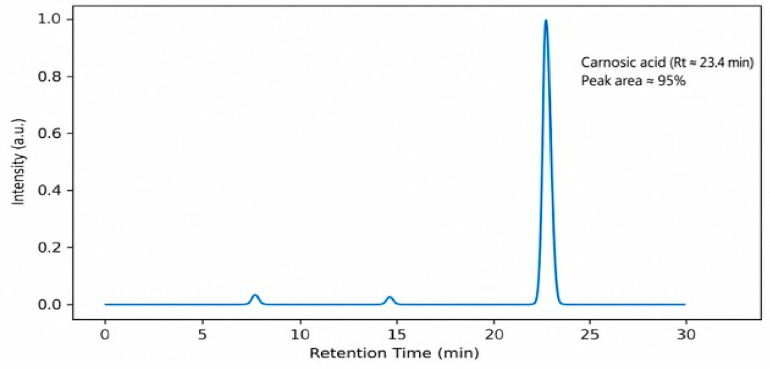
UHPLC–QTOF-MS chromatogram of the purified fraction showing a single dominant peak of carnosic acid (Rt ≈ 23.4 min). Purity (>95%) was determined by LC peak area normalization relative to the total integrated chromatographic area.

Thin-layer chromatography was employed as a rapid, cost-effective, and robust analytical technique for the preliminary phytochemical screening of the methanolic extract of *R. officinalis* leaves, as well as for the verification of carnosic acid presence and its effective chromatographic separation. Separation was performed on silica gel 60 F254 plates (20 × 20 cm) using a mobile phase composed of n-hexane:ethyl acetate (8:2, *v*/*v*), a solvent system widely validated for the efficient resolution of lipophilic abietane-type diterpenes in rosemary-derived matrices (Figure 2). The TLC fingerprint of the crude methanolic extract (Lane 1) revealed multiple well-defined and clearly resolved chromatographic bands exhibiting diverse chromogenic responses, including brown, blue, green, orange, and yellow spots. This pattern reflects the chemical heterogeneity of the extract and indicates the coexistence of multiple phytochemical classes, including phenolic acids, flavonoids, and diterpenoid constituents. Importantly, a distinct yellow band observed in the extract lane migrated with an identical retention factor (Rf = 0.62) and showed perfect co-localization with the authentic carnosic acid reference standard (Lane 2). The concordance in both Rf value and visual band characteristics provides strong qualitative chromatographic evidence supporting the presence of carnosic acid within the methanolic extract. The silica gel region corresponding to the carnosic acid band was carefully excised and subjected to elution with methanol to recover the adsorbed compound. Sequential TLC-based fractionation of 1.0 g of crude methanolic extract enabled efficient enrichment of the target analyte, yielding approximately 73 mg/g (~7.3% recovery). Scaling the process to a total of 5.0 g of crude extract resulted in the isolation of approximately 365 mg of carnosic acid. The isolated fraction was subsequently analyzed by UHPLC/QTOF-MS, which confirmed its structural identity and high analytical purity based on accurate mass determination, chromatographic retention behavior, and diagnostic spectral features. The purified compound exhibited a single, sharp, and well-resolved chromatographic peak at Rt ≈ 23.4 min (Figure 3), thereby confirming the successful isolation and high-purity enrichment of carnosic acid.

### 3.4. Antibacterial Activity of Carnosic Acid and MICs

The antibacterial efficacy of carnosic acid was evaluated against a panel of reference and foodborne bacterial strains and systematically compared with the fluoroquinolone antibiotic ciprofloxacin (Table 4). Overall, carnosic acid demonstrated moderate to strong antibacterial activity, as reflected by inhibition zone diameters (IZDs) ranging from 12.6 to 23.8 mm and MICs spanning 10–23 µg/mL, depending on the tested organism. In contrast, ciprofloxacin consistently exhibited superior antimicrobial potency, as evidenced by significantly larger inhibition zones and substantially lower MIC values across all bacterial strains evaluated.

Statistical analysis confirmed that the differences in IZDs between carnosic acid and ciprofloxacin were highly significant for all tested microorganisms (*p* < 0.01–0.001), thereby substantiating the greater in vitro efficacy of the reference antibiotic under the applied experimental conditions. Notably, carnosic acid displayed appreciable activity against foodborne pathogenic bacteria, including *Listeria monocytogenes*, *Staphylococcus aureus*, and *Escherichia coli*, with inhibition zones ranging from 15.8 to 20.2 mm.

Importantly, carnosic acid exhibited bactericidal properties, as indicated by MBC/MIC ratios of approximately 2 across all tested strains. This indicates that the compound not only suppresses bacterial growth but also exerts lethal effects at concentrations close to its inhibitory threshold. Such a narrow transition between inhibitory and bactericidal concentrations is particularly favorable, as it may reduce the likelihood of bacterial persistence and potentially limit the emergence of resistance.

The observed bactericidal activity was consistent across both Gram-positive bacteria (*Staphylococcus aureus*, *Bacillus subtilis*, *Listeria monocytogenes*, *Enterococcus faecalis*) and Gram-negative bacteria (*Escherichia coli*, *Klebsiella pneumoniae*, *Salmonella Typhimurium*, *Pseudomonas aeruginosa*), underscoring the broad-spectrum antimicrobial potential of carnosic acid.

### 3.5. Antibiofilm Activity of Carnosic Acid

**Figure 4:**
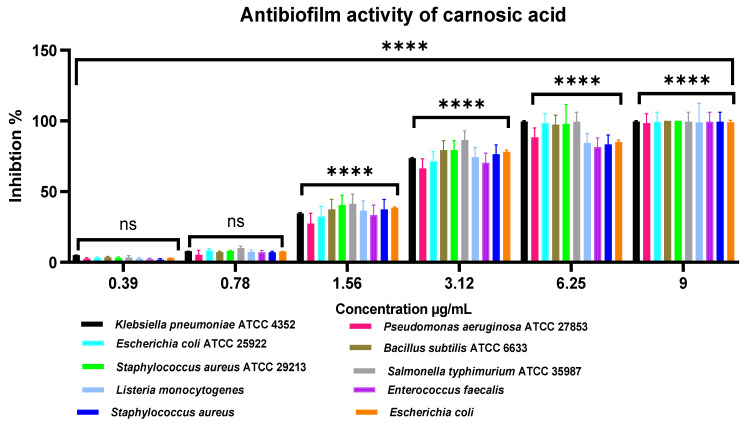
Antibiofilm activity of purified carnosic acid against selected bacterial strains, determined using the crystal violet microtiter plate assay. Biofilm formation was quantified after 24 h of incubation with increasing concentrations of carnosic acid and expressed as percentage inhibition relative to untreated controls. Data are presented as mean ± SD (*n* = 3), **** *p* < 0.0001; ns, not significant *p* ≥ 0.05.

The antibiofilm activity of carnosic acid was evaluated against a representative panel of Gram-positive and Gram-negative bacterial strains at sub-inhibitory concentrations ranging from 0.39 to 9.0 µg/mL (Figure 4). Overall, carnosic acid demonstrated a clear concentration-dependent inhibitory effect on biofilm formation across all tested microorganisms.

At the lowest concentration (0.39 µg/mL), only negligible antibiofilm activity was observed, with inhibition rates remaining below 5% for all strains. A marginal increase in activity was detected at 0.78 µg/mL; however, biofilm inhibition remained limited (<15%), indicating minimal efficacy at very low exposure levels. A more pronounced enhancement in antibiofilm activity was observed at 1.56 µg/mL, where inhibition values ranged approximately from 25% to 45%, depending on the bacterial species.

A substantial escalation in activity was recorded at 3.12 µg/mL, at which point biofilm inhibition exceeded 60% for most tested strains, including *Klebsiella pneumoniae* ATCC 4352, *Pseudomonas aeruginosa* ATCC 27853, *Staphylococcus aureus* ATCC 29213, and *Escherichia coli* ATCC 25922. At higher concentrations (6.25 and 9.0 µg/mL), carnosic acid induced near-complete suppression of biofilm formation, with inhibition rates approaching or surpassing 90% across all bacterial species examined. Notably, both reference and foodborne isolates of *Staphylococcus aureus* and *Escherichia coli*, as well as intrinsically robust biofilm-forming organisms such as *Pseudomonas aeruginosa* and *Enterococcus faecalis*, exhibited marked susceptibility at these higher concentrations.

According to the statistical annotations shown in Figure 4, biofilm inhibition increased significantly in a concentration-dependent manner across all tested bacterial strains. The lowest concentrations (0.39 and 0.78 μg/mL) produced minimal inhibition and did not differ significantly from each other (*p* > 0.05). In contrast, concentrations from 1.56 μg/mL onward resulted in progressively greater antibiofilm activity, with highly significant increases observed between successive concentration levels (*p* < 0.0001). The highest inhibition percentages were achieved at 6.25 and 9 μg/mL, where biofilm suppression approached 90–100% in several strains. Although strain-specific differences were evident, all tested organisms exhibited a similar dose-dependent response to carnosic acid.

### 3.6. Antioxidant Activity of Carnosic Acid Assessed by DPPH and ABTS Assays

The antioxidant capacity of carnosic acid was systematically evaluated in comparison with ascorbic acid over a concentration range of 7.81–1000 µg/mL using both DPPH and ABTS radical scavenging assays (Table 5). In both systems, a pronounced concentration-dependent increase in radical scavenging activity was observed for carnosic acid, indicating progressive enhancement of its antioxidant efficacy with increasing dose.

In the DPPH assay, carnosic acid exhibited a gradual elevation in free radical scavenging activity from 12.3 ± 0.5% to 92.1 ± 2.0% across the tested concentration range. In parallel, ascorbic acid consistently demonstrated higher radical neutralization capacity, achieving a maximum inhibition of 97.4 ± 2.0% at the highest concentration. Correspondingly, IC_50_ analysis revealed a significantly lower value for ascorbic acid (88 ± 4 µg/mL) compared with carnosic acid (125 ± 5 µg/mL; *p* < 0.05), indicating superior antioxidant potency of the reference compound, likely attributable to its higher electron-donating efficiency and intrinsic redox reactivity.

A comparable trend was observed in the ABTS assay, where carnosic acid demonstrated strong radical scavenging activity, reaching 98.8 ± 1.9% inhibition at the highest concentration tested, closely approaching that of ascorbic acid (96.3 ± 1.9%). Nonetheless, IC_50_ determination again confirmed greater potency for ascorbic acid (95 ± 5 µg/mL) relative to carnosic acid (130 ± 6 µg/mL; *p* < 0.05).

Overall, statistical analysis confirmed significant differences in radical scavenging activity between carnosic acid and ascorbic acid across all tested concentrations (*p* < 0.05), despite the substantial and biologically relevant antioxidant capacity exhibited by carnosic acid in both assay systems.

### 3.7. Anti-Inflammatory Activity of Carnosic Acid Assessed by Human Red Blood Cell Membrane Stabilization

The anti-inflammatory potential of carnosic acid was evaluated in comparison with sodium diclofenac using the human red blood cell (HRBC) membrane stabilization assay. This in vitro model assesses the ability of test compounds to inhibit heat-induced erythrocyte hemolysis and is widely recognized as a surrogate indicator of anti-inflammatory activity. Stabilization of the erythrocyte membrane is mechanistically correlated with lysosomal membrane stabilization, thereby contributing to the attenuation of the release of pro-inflammatory enzymes and mediators under conditions of cellular stress.

Both carnosic acid and sodium diclofenac exhibited a clear concentration-dependent inhibition of hemolysis, indicating progressive enhancement of membrane-stabilizing capacity with increasing concentrations. Nevertheless, sodium diclofenac consistently demonstrated significantly greater protective efficacy than carnosic acid across all tested concentrations (*p* < 0.05; Table 6). Correspondingly, IC_50_ analysis revealed values of 94.3 µg/mL for carnosic acid and 64.2 µg/mL for sodium diclofenac, confirming the superior potency of the reference anti-inflammatory drug under the experimental conditions.

### 3.8. Anti-Diabetic Activity of Carnosic Acid

**Figure 5:**
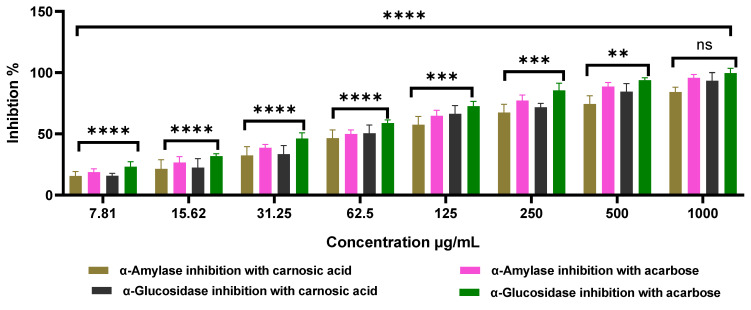
Anti-diabetic activity of purified carnosic acid. The inhibitory effects of purified carnosic acid against α-amylase and α-glucosidase were evaluated at concentrations ranging from 7.81 to 1000 µg/mL and compared with the standard inhibitor acarbose. Data are presented as mean ± SD (*n* = 3), ** *p* < 0.01, *** *p* < 0.001, **** *p* < 0.0001; ns, not significant *p* ≥ 0.05.

The antidiabetic potential of carnosic acid was evaluated through its inhibitory effects on two key carbohydrate-hydrolyzing enzymes, α-amylase and α-glucosidase, which play central roles in postprandial glucose regulation (Figure 5). Carnosic acid exhibited a clear concentration-dependent inhibitory profile against both enzymes over the tested range (7.81–1000 µg/mL), with statistically significant enhancement of enzyme inhibition observed at increasing concentrations.

At higher concentrations (500–1000 µg/mL), α-amylase inhibition exceeded approximately 70%, whereas α-glucosidase inhibition reached nearly 90%, indicating pronounced suppression of carbohydrate-digesting enzymatic activity. Notably, α-glucosidase inhibition was consistently and significantly greater than α-amylase inhibition at corresponding concentrations, suggesting a preferential inhibitory selectivity toward α-glucosidase.

In comparison with the reference inhibitor acarbose, carnosic acid exhibited comparatively lower inhibitory activity at low and intermediate concentrations; however, at higher concentrations, its activity approached levels comparable to acarbose, particularly in the case of α-glucosidase inhibition.

Statistical analysis revealed a significant concentration-dependent increase in both α-amylase and α-glucosidase inhibition following treatment with carnosic acid and acarbose (*p* < 0.05). Multiple-comparison analysis demonstrated significant differences among most concentration levels, with inhibitory activity progressively increasing from 7.81 to 500 μg/mL. However, no significant difference was observed between the highest concentration (1000 μg/mL) and the preceding concentration, indicating saturation of enzyme inhibition at elevated doses (ns, *p* > 0.05). Overall, carnosic acid exhibited a dose-responsive inhibitory effect against both enzymes, approaching the activity of the reference inhibitor acarbose at higher concentrations (Figure 5). Overall, the results demonstrate a progressive, dose-dependent enhancement of both α-amylase and α-glucosidase inhibition by carnosic acid, with maximal inhibitory effects observed at 1000 µg/mL.

### 3.9. Cytotoxic and Antiproliferative Effects of Carnosic Acid

The anticancer activity of carnosic acid was evaluated against human breast adenocarcinoma (MCF-7), hepatocellular carcinoma (HepG2), and normal human breast epithelial (MCF-10A) cell lines using a standard cell viability assay. As presented in Table 7, carnosic acid induced a clear concentration-dependent decrease in cellular viability across all evaluated cell lines; however, the cytotoxic response was markedly more pronounced in cancerous cells relative to non-tumorigenic epithelial cells.

At lower concentrations (6.25–12.5 µg/mL), no statistically significant differences in viability were observed between MCF-7 and HepG2 cells (*p* > 0.05), whereas MCF-10A cells maintained significantly higher viability, indicating an inherent resistance of normal epithelial cells to carnosic acid exposure. At 25 µg/mL, a statistically significant reduction in viability was observed in MCF-7 cells (61.2 ± 2.3%) compared with HepG2 cells (68.5 ± 2.4%) (*p* = 0.032). In parallel, both malignant cell lines exhibited significantly lower viability relative to MCF-10A cells (88.7 ± 1.6%; *p* < 0.0001), confirming a differential cytotoxic response favoring tumor cells. This selective cytotoxic pattern was consistently maintained across increasing concentrations.

At 250 µg/mL, viability decreased substantially to 10.8 ± 1.3% in MCF-7 cells and 16.9 ± 1.5% in HepG2 cells, whereas MCF-10A cells retained 70.4 ± 1.8% viability, further substantiating the tumor-selective cytotoxic profile of carnosic acid. At concentrations ≥100 µg/mL, cell viability remained below 45% in both cancer cell lines, while exceeding 70% in normal MCF-10A cells, reinforcing the preferential toxicity toward malignant phenotypes.

Moreover, MCF-7 cells demonstrated consistently greater sensitivity to carnosic acid than HepG2 cells, as evidenced by lower viability values and a significantly reduced IC_50_ (28.3 ± 1.2 µg/mL) compared with HepG2 cells (37.8 ± 1.4 µg/mL). In contrast, the IC_50_ value for MCF-10A cells exceeded 250 µg/mL, indicating markedly reduced susceptibility of normal breast epithelial cells. To further quantify tumor selectivity, the selectivity index (SI) was calculated as IC_50_ (MCF-10A)/IC_50_ (cancer cells). The resulting SI values were >8.8 for MCF-7 cells and >6.6 for HepG2 cells, confirming a strong preferential cytotoxic effect of carnosic acid toward malignant cells over normal epithelial counterparts.

### 3.10. Modulation of Apoptosis-Associated Gene Expression by Carnosic Acid

Apoptosis represents a tightly regulated physiological process responsible for the selective removal of damaged, dysfunctional, or malignant cells, and its impairment is widely recognized as a critical hallmark of tumor initiation, progression, and therapeutic resistance. In the present study, the regulatory effects of carnosic acid on apoptosis-associated gene expression were investigated through quantitative assessment of the pro-apoptotic gene *BAX* and the anti-apoptotic gene *BCL2* in human breast adenocarcinoma (MCF-7) and hepatocellular carcinoma (HepG2) cell lines. Gene expression levels were normalized against untreated controls and expressed as relative fold change.

In MCF-7 cells, *BAX* expression increased from a baseline value of approximately 1.0-fold in untreated controls to ~2.7-fold following carnosic acid treatment, and further to ~3.5-fold after exposure to doxorubicin. Statistically significant differences were observed between untreated and carnosic acid-treated cells (*p* < 0.001), untreated and doxorubicin-treated cells (*p* < 0.0001), as well as between carnosic acid- and doxorubicin-treated groups (*p* < 0.05). In parallel, *BCL2* expression exhibited a marked downregulation, decreasing from ~1.0-fold in control cells to ~0.5-fold following carnosic acid treatment and further declining to ~0.2–0.3-fold under doxorubicin exposure. These changes were statistically significant across all comparisons, including untreated versus carnosic acid-treated cells (*p* < 0.001), untreated versus doxorubicin-treated cells (*p* < 0.0001), and carnosic acid versus doxorubicin treatments (*p* < 0.05).

**Figure 6:**
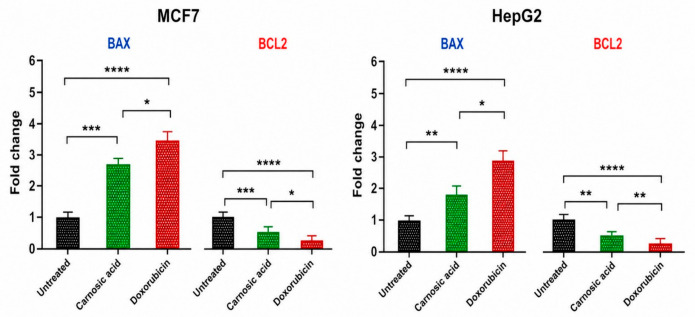
Relative expression levels of *BAX* and *BCL2* in MCF-7 and HepG2 cells following treatment with carnosic acid (25 μg/mL) or doxorubicin (5 μg/mL) compared with untreated controls. Data are presented as mean ± SD from three independent biological replicates (*n* = 3) and are expressed as fold change relative to the untreated control, * *p* < 0.05, ** *p* < 0.01, *** *p* < 0.001, **** *p* < 0.0001; ns, not significant *p* ≥ 0.05.

A comparable expression pattern was observed in HepG2 cells. BAX expression increased from ~1.0-fold in untreated cells to ~1.8-fold following carnosic acid exposure and to ~2.9-fold after doxorubicin treatment. Significant differences were identified between untreated and carnosic acid-treated groups (*p* < 0.01), untreated and doxorubicin-treated cells (*p* < 0.0001), and between the two treatment conditions (*p* < 0.05). Conversely, BCL2 expression was reduced to approximately 0.5-fold following carnosic acid treatment and further decreased to ~0.2–0.3-fold under doxorubicin exposure, with statistically significant differences observed between all comparative groups (untreated vs. carnosic acid, *p* < 0.01; untreated vs. doxorubicin, *p* < 0.0001; and carnosic acid vs. doxorubicin, *p* < 0.01), as illustrated in Figure 6.

## 4. Discussion

Plant-derived natural products have served as a cornerstone of human health and wellness for millennia and continue to play a pivotal role in the pharmaceutical, food, and cosmetic industries. Across diverse civilizations, medicinal plants have traditionally been utilized not only as dietary resources but also as therapeutic agents for the prevention and management of numerous diseases [1,3]. In recent decades, growing interest in natural bioactive compounds, coupled with advances in analytical and omics technologies, has accelerated the scientific exploration of medicinal plants as valuable sources of structurally diverse and pharmacologically active metabolites. Consequently, considerable research efforts have been directed toward elucidating their phytochemical composition, biological activities, and potential applications in drug discovery, functional foods, nutraceuticals, and cosmetic formulations [5,6,7,8,9].

In this context, the present study integrated UHPLC/QTOF-MS-based phytochemical profiling of *R. officinalis* with the isolation and characterization of carnosic acid, one of its principal bioactive diterpenes. Furthermore, the study evaluated the selective α-glucosidase inhibitory activity and sub-MIC antibiofilm efficacy of the isolated compound. Collectively, the findings provide additional insight into the multifunctional pharmacological potential of carnosic acid and contribute to the growing body of evidence supporting its development as a promising natural bioactive agent.

The present study highlights the critical influence of solvent polarity on the extraction efficiency and phytochemical composition of *R. officinalis* leaves. Methanol and ethanol yielded significantly higher extraction recoveries and enriched concentrations of phenolics, flavonoids, tannins, saponins, alkaloids, and terpenoids compared with less polar solvents, confirming the superior capacity of polar solvents to solubilize and recover bioactive secondary metabolites. These observations are consistent with previous reports demonstrating enhanced extraction of polyphenolic compounds and other phytochemicals using polar organic solvents due to their favorable polarity and hydrogen-bonding interactions [7,29,31,32].

Comprehensive UHPLC/QTOF-MS profiling revealed a chemically complex metabolome dominated by phenolic acids, flavonoids, and diterpenoids. Among the major constituents identified were rosmarinic acid, caffeic acid derivatives, luteolin and apigenin glycosides, carnosic acid, and carnosol, all of which have been extensively associated with the antioxidant, antimicrobial, anti-inflammatory, and anticancer properties of rosemary [4,30,33,34,35,36,37,38]. The predominance of carnosic acid and carnosol in the methanolic extract corroborates previous studies identifying these abietane diterpenes as principal bioactive markers of *R. officinalis* [29]. Furthermore, the simultaneous extraction of hydrophilic phenolics and moderately lipophilic diterpenoids underscores the suitability of methanol as a solvent for comprehensive phytochemical recovery [32,35].

Purification of carnosic acid by TLC followed by UHPLC/QTOF-MS confirmation yielded a highly purified fraction (>95%), validating the effectiveness of the applied isolation strategy. The chromatographic behavior and recovery efficiency were consistent with previous reports describing the successful separation of abietane diterpenes using silica-based chromatographic systems [39,40,41]. Given its relatively high abundance and broad spectrum of biological activities, carnosic acid was selected as the primary target compound for further investigation [42,43,44].

The isolated carnosic acid exhibited pronounced antibacterial activity against both Gram-positive and Gram-negative bacteria, although its efficacy remained lower than that of ciprofloxacin. The greater susceptibility of Gram-positive organisms is likely attributable to the absence of an outer lipopolysaccharide membrane, which facilitates the penetration of hydrophobic compounds such as carnosic acid [7,41,45,46]. The observed bactericidal activity is consistent with reports indicating that phenolic diterpenes compromise membrane integrity, increase cellular permeability, and disrupt essential metabolic processes [47,48]. Unlike conventional antibiotics that act on specific molecular targets, carnosic acid appears to exert a multifaceted mode of action, potentially reducing the risk of resistance development and enhancing its value as an adjunct antimicrobial agent [49]. Its effectiveness against important foodborne pathogens, including *Listeria monocytogenes*, *Staphylococcus aureus*, and *Escherichia coli*, further supports its potential application in food preservation and safety systems [50,51].

Biofilm-associated infections represent a significant clinical and industrial challenge because biofilm-embedded microorganisms exhibit enhanced tolerance to antimicrobial agents. In the present study, carnosic acid produced potent concentration-dependent inhibition of biofilm formation, with near-complete suppression observed at concentrations of 6.25–9.0 μg/mL. These findings are in agreement with previous studies demonstrating that phenolic diterpenes interfere with bacterial adhesion, quorum sensing, extracellular polymeric substance synthesis, and biofilm maturation [47,48,52]. The pronounced activity against *Staphylococcus aureus* and *Pseudomonas aeruginosa*, two clinically important biofilm-forming pathogens, is particularly noteworthy and highlights the potential of carnosic acid as a natural antibiofilm agent for biomedical and food industry applications [48,53,54,55].

Carnosic acid also demonstrated substantial antioxidant activity in both DPPH and ABTS assays, although its potency was lower than that of ascorbic acid. This antioxidant capacity is primarily attributed to its ortho-diphenolic structure, which facilitates efficient electron donation and radical scavenging [7,56,57]. Previous investigations have shown that carnosic acid accounts for the majority of rosemary’s antioxidant activity and effectively suppresses lipid peroxidation and reactive oxygen species generation [57,58]. Additionally, synergistic interactions among carnosic acid, carnosol, and rosmarinic acid may further amplify the overall antioxidant efficacy of rosemary extracts [59]. The strong antioxidant properties observed herein likely contribute to the diverse biological activities demonstrated by the compound.

The anti-inflammatory potential of carnosic acid was further confirmed through the HRBC membrane stabilization assay. Although sodium diclofenac exhibited greater potency, carnosic acid significantly protected erythrocyte membranes against hemolysis in a concentration-dependent manner. Since erythrocyte membrane stabilization is considered analogous to lysosomal membrane stabilization, these findings suggest the ability of carnosic acid to limit the release of pro-inflammatory mediators during tissue injury. Mechanistically, previous studies have demonstrated that carnosic acid suppresses inflammatory responses through inhibition of NF-κB, STAT3, PI3K/Akt, and MAPK signaling pathways, leading to reduced expression of inflammatory cytokines and mediators [7,60,61,62,63]. A key limitation of this study is that the HRBC membrane stabilization assay was performed using blood from a single healthy donor (biological *n* = 1), which may limit the generalizability of the findings.

The antidiabetic assessment revealed significant concentration-dependent inhibition of both α-amylase and α-glucosidase, with a clear preference toward α-glucosidase inhibition. This selective activity is therapeutically advantageous because it can attenuate postprandial hyperglycemia while minimizing the gastrointestinal side effects commonly associated with excessive α-amylase inhibition. These findings align with previous studies demonstrating that carnosic acid modulates carbohydrate metabolism through direct enzyme inhibition and regulation of glucose homeostasis [50,64]. Moreover, clinical investigations reporting improvements in glycemic control, glycated hemoglobin, and insulin sensitivity following rosemary supplementation provide additional support for the translational relevance of these observations [65,66,67].

A particularly significant finding of the present study was the selective cytotoxicity of carnosic acid toward MCF-7 and HepG2 cancer cells, while exerting minimal toxicity toward normal MCF-10A cells. The lower IC_50_ values observed in malignant cells indicate preferential targeting of cancerous tissues, an essential characteristic of prospective anticancer agents. These results are consistent with previous reports demonstrating that carnosic acid suppresses tumor cell proliferation through induction of cell-cycle arrest, oxidative stress modulation, and apoptosis mediated by PI3K/Akt, MAPK, and NF-κB signaling pathways [14,68,69]. The greater sensitivity of MCF-7 cells may be attributable to differences in metabolic activity, hormonal responsiveness, and antioxidant defense mechanisms between breast and hepatic cancer cells [70].

Mechanistic insights were further provided by the analysis of apoptosis-related genes. Treatment with carnosic acid significantly upregulated the pro-apoptotic gene *BAX* while downregulating the anti-apoptotic gene *BCL2*, resulting in a marked increase in the *BAX/BCL2* ratio in both cancer cell lines. This ratio is a well-established indicator of mitochondrial apoptotic pathway activation and is closely associated with cytochrome c release, caspase activation, and programmed cell death [71,72,73,74]. Previous studies have shown that carnosic acid promotes apoptosis through reactive oxygen species generation, mitochondrial dysfunction, and modulation of p53, PI3K/Akt, and MAPK signaling pathways [14,75,76,77,78,79]. The stronger transcriptional response observed in MCF-7 cells may reflect differences in mitochondrial priming and hormone-dependent signaling pathways characteristic of breast cancer biology [80].

Collectively, the present findings demonstrate that carnosic acid is a multifunctional bioactive diterpene possessing antioxidant, antibacterial, antibiofilm, anti-inflammatory, antidiabetic, and anticancer activities. The integration of comprehensive phytochemical characterization with functional and molecular analyses provides robust evidence supporting its therapeutic potential. Nevertheless, further in vivo studies, pharmacokinetic investigations, and protein-level mechanistic analyses are required to validate these findings and facilitate the development of carnosic acid as a natural therapeutic agent and functional bioactive ingredient.

## 5. Conclusions

This study provides comprehensive evidence supporting the therapeutic potential of *R. officinalis* L., with particular emphasis on carnosic acid as a prominent multifunctional bioactive constituent. Comprehensive UHPLC/QTOF-MS-based metabolomic profiling revealed a chemically diverse phytochemical landscape enriched in phenolic acids, flavonoids, and abietane diterpenes, enabling the targeted identification, isolation, and purification of carnosic acid. The purified compound exhibited broad-spectrum antibacterial activity, with pronounced efficacy against Gram-positive pathogens and potent inhibition of biofilm formation at sub-minimum inhibitory concentrations. Beyond its antimicrobial properties, carnosic acid demonstrated substantial antioxidant and anti-inflammatory activities, selective inhibition of α-glucosidase relative to α-amylase, and significant antiproliferative effects against MCF-7 and HepG2 cancer cells. Notably, the upregulation of *BAX* coupled with the downregulation of *BCL2* resulted in a marked increase in the *BAX/BCL2* ratio, indicating activation of the intrinsic mitochondrial apoptotic pathway. Collectively, these findings establish carnosic acid as a promising multi-target phytochemical with potential applications in antimicrobial intervention, metabolic disease management, and cancer therapy. However, the present study is limited to in vitro experimental models, including MTT-based cytotoxicity assays and mRNA expression analysis, without validation at the protein level or confirmation of downstream signaling pathways. In addition, the absence of in vivo studies limits the ability to extrapolate these findings to physiological conditions, particularly with regard to bioavailability, pharmacokinetics, and systemic toxicity. Therefore, further in vivo investigations, pharmacokinetic profiling, and comprehensive mechanistic studies are required to substantiate its clinical relevance and support its development as a natural therapeutic agent or functional bioactive ingredient.

## Figures and Tables

**Table 1:** Primer sequences for *BAX*, *BCL2*, and *GAPDH*.

Gene	Forward Primer	Reverse Primer
***BAX***	CGAACTGGACAGTAACATGG	CAGTTTGCTGGCAAAGTAGA
***BCL2***	GATTGTGGCCTTCTTTGAGT	ATAGGCACCCAGGGTGAT
***GAPDH***	GAAGGTGAAGGTCGGAGTCA	GAGATGGTGATGGGATTTC

**Table 2:** Quantitative phytochemical composition of *R. officinalis* leaves extracts.

Phytochemical	Hexane	Ethyl Acetate	Methanol	Ethanol
Saponins (mg/g DW)	12.5 ± 0.5 ^c^	21.0 ± 0.8 ^b^	35.0 ± 1.2 ^a^	32.0 ± 1.0 ^a^
Alkaloids (mg/g DW)	8.0 ± 0.3 ^c^	15.0 ± 0.5 ^b^	27.5 ± 1.0 ^a^	25.0 ± 0.8 ^a^
Tannins (mg TAE/g DW)	9.5 ± 0.4 ^c^	18.0 ± 0.7 ^b^	32.0 ± 1.1 ^a^	30.0 ± 0.9 ^a^
Flavonoids (mg QE/g DW)	11.0 ± 0.5 ^c^	22.5 ± 0.9 ^b^	40.0 ± 1.3 ^a^	38.0 ± 1.2 ^a^
Total Phenols (mg GAE/g DW)	15.0 ± 0.6 ^c^	28.0 ± 1.0 ^b^	45.0 ± 1.5 ^a^	42.0 ± 1.3 ^a^
Steroids (mg CE/g DW)	7.0 ± 0.3 ^c^	12.0 ± 0.5 ^b^	21.0 ± 0.9 ^a^	19.5 ± 0.8 ^a^
Terpenoids (mg/g DW)	14.0 ± 0.5 ^c^	25.0 ± 0.9 ^b^	41.0 ± 1.4 ^a^	39.0 ± 1.2 ^a^

Values are mean ± SD (*n* = 3). Different letters within the same row indicate significant differences (*p* < 0.05) according to one-way ANOVA followed by Tukey’s post hoc test. DW, dry weight; TAE, tannic acid equivalents; QE, quercetin equivalents; GAE, gallic acid equivalents; CE, cholesterol equivalents.

**Table 3:** Summary of major metabolites identified in *R. officinalis* methanolic extract by UHPLC/QTOF-MS.

Peak No.	Retention Time (min)	Tentative Identification	Compound Class	Molecular Ion (*m*/*z*)	Molecular Formula	Biological Relevance	References
1	4.8–5.6	Rosmarinic acid	Phenolic acid	359.077 [M−H]^−^	C_18_H_16_O_8_	Potent antioxidant, anti-inflammatory	[3,4,5,6,7,8,29,30]
2	6.5–8.2	Caffeic acid derivatives	Hydroxycinnamic acids	179.035/341.066 [M−H]^−^	C_9_H_8_O_4_/C_18_H_16_O_8_	Antioxidant, antimicrobial	[3,4,5,6,7,8,29,30]
3	13.2–14.8	Luteolin glycosides	Flavonoids	447.093 [M−H]^−^	C_21_H_20_O_11_	Anti-inflammatory, anticancer	[3,4,5,6,7,8,29,30]
4	15.1–16.7	Apigenin derivatives	Flavonoids	269.045 [M−H]^−^	C_15_H_10_O_5_	Antioxidant, enzyme inhibition	[3,4,5,6,7,8,29,30]
5	23.4–24.2	Carnosic acid	Phenolic diterpene	331.191 [M−H]^−^	C_20_H_28_O_4_	Antioxidant, anticancer, neuroprotective	[3,4,5,6,7,8,29,30]
6	24.8–25.6	Carnosol	Phenolic diterpene	329.176 [M−H]^−^	C_20_H_26_O_4_	Anti-inflammatory, pro-apoptotic	[3,4,5,6,7,8,29,30]
7	26.0–27.4	Abietane diterpenes	Diterpenoids	301–345 [M−H]^−^	C_20_H_30_O_2_–_4_	Antimicrobial, antioxidant	[3,4,5,6,7,8,29,30]

**Table 4:** Antibacterial activity of carnosic acid and ciprofloxacin.

Bacterial Strains	Carnosic Acid				Ciprofloxacin		Statistical Comparison *p*-Value
	IZD (mm)	MIC (µg/mL)	MBC (µg/mL)	MBC/MIC	IZD (mm)	MIC (µg/mL)	
*Klebsiella pneumoniae* ATCC 4352	21.4 ± 1.2	16	32	2	28.6 ± 1.1	0.25	<0.001
*Pseudomonas aeruginosa* ATCC 27853	23.8 ± 1.3	18	36	2	26.9 ± 1.0	0.50	<0.001
*Escherichia coli ATCC* 25922	15.6 ± 1.1	12	24	2	30.2 ± 1.3	0.06	<0.001
*Bacillus subtilis* ATCC 6633	16.9 ± 1.2	10	20	2	32.4 ± 1.2	0.12	<0.001
*Staphylococcus aureus* ATCC 29213	14.8 ± 0.9	10	20	2	33.1 ± 1.4	0.25	<0.001
*Salmonella typhimurium* ATCC 35987	12.6 ± 0.8	11	22	2	27.5 ± 1.1	0.12	<0.001
*Listeria monocytogenes*	20.2 ± 1.0	16	32	2	31.8 ± 1.3	0.50	<0.01
*Enterococcus faecalis*	18.4 ± 0.9	23	46	2	29.4 ± 1.2	1.00	<0.001
*Staphylococcus aureus*	19.7 ± 1.1	13	26	2	30.6 ± 1.5	0.50	<0.001
*Escherichia coli*	15.8 ± 1.0	14	28	2	27.1 ± 1.0	0.12	<0.001

IZD: inhibition zone diameter (mm); MIC: minimum inhibitory concentration; MBC: minimum bactericidal concentration. Values are expressed as mean ± SD (*n* = 3).

**Table 5:** Antioxidant activity of carnosic acid evaluated by DPPH and ABTS assays.

Concentration (µg/mL)	DPPH Scavenging (%)		ABTS Scavenging (%)	
	Carnosic Acid	Ascorbic Acid	Carnosic Acid	Ascorbic Acid
7.81	12.3 ± 0.5	14.1 ± 0.7 *	10.5 ± 0.6	13.2 ± 0.5 *
15.62	20.8 ± 0.7	23.5 ± 0.8 *	18.7 ± 0.9	21.6 ± 0.8 *
31.25	33.4 ± 1.0	37.8 ± 1.2 *	30.1 ± 1.0	35.5 ± 1.1 *
62.5	46.9 ± 1.3	52.2 ± 1.5 *	44.0 ± 1.2	50.8 ± 1.4 *
125	60.7 ± 1.5	68.3 ± 1.7 *	58.1 ± 1.5	65.0 ± 1.6 *
250	73.2 ± 1.6	81.0 ± 1.8 *	71.0 ± 1.7	78.5 ± 1.7 *
500	85.5 ± 1.8	91.5 ± 1.9 *	83.6 ± 1.8	89.7 ± 1.8 *
1000	92.1 ± 2.0	97.4 ± 2.0 *	98.8 ± 1.9	96.3 ± 1.9 *
IC_50_ (μg/mL)	125 ± 5	88 ± 4 *	130 ± 6	95 ± 5 *

Data are presented as mean ± standard deviation (SD) (*n* = 3). DPPH and ABTS radical scavenging activities were determined at the indicated concentrations. Statistical significance was analyzed using appropriate comparative tests; values marked with an asterisk (*) indicate a significant difference compared with carnosic acid at the corresponding concentration (*p* < 0.05). IC_50_ values represent the concentration required to achieve 50% radical scavenging activity.

**Table 6:** Anti-inflammatory activity of carnosic acid.

Concentration (µg/mL)	% Inhibition of Hemolysis, Mean ± SD		*p*-Value
	Carnosic Acid	Sodium Diclofenac	
**7.81**	8.5 ± 0.6	12.1 ± 0.7	0.032
**15.62**	15.2 ± 0.8	21.5 ± 1.0	0.018
**31.25**	26.4 ± 1.2	33.8 ± 1.3	0.012
**62.5**	41.0 ± 1.5	49.2 ± 1.6	0.009
**125**	58.7 ± 1.8	65.5 ± 1.9	0.006
**250**	72.4 ± 2.0	78.1 ± 2.1	0.004
**500**	84.3 ± 2.2	89.5 ± 2.3	0.003
**1000**	91.6 ± 2.4	95.2 ± 2.5	0.002
IC_50_ (µg/mL)	94.3	64.2	-----

Values are expressed as mean ± SD (*n* = 3). Statistical comparisons between carnosic acid and the standard anti-inflammatory drug (sodium diclofenac) at equivalent concentrations were performed using an unpaired Student’s *t*-test.

**Table 7:** Anti-cancer activity of carnosic acid.

Concentration (µg/mL)	MCF7 Cell Viability (%)	HepG2 Cell Viability (%)	MCF-10A Cell Viability (%)	*p*-Value (MCF7 vs. MCF-10A)	*p*-Value (HepG2 vs. MCF-10A)
6.25	86.8 ± 1.5	88.2 ± 1.6	95.3 ± 2.1	<0.01	<0.05
12.5	79.6 ± 2.1	81.0 ± 2.0	91.0 ± 1.0	<0.001	<0.001
25	61.2 ± 2.3	68.5 ± 2.4	88.7 ± 1.6	<0.0001	<0.0001
50	49.3 ± 2.0	56.8 ± 2.1	82.4 ± 1.4	<0.0001	<0.0001
100	37.5 ± 1.9	44.2 ± 2.0	80.4 ± 2.1	<0.0001	<0.0001
150	29.4 ± 1.8	37.8 ± 2.2	75.7 ± 1.2	<0.0001	<0.0001
200	20.1 ± 1.6	25.4 ± 1.9	73.2 ± 1.4	<0.0001	<0.0001
250	10.8 ± 1.3	16.9 ± 1.5	70.4 ± 1.8	<0.0001	<0.0001

Values are expressed as mean ± SD (*n* = 3).

## Data Availability

The original contributions presented in this study are included in the article. Further inquiries can be directed to the corresponding author.
